# Extracellular matrix turnover biomarkers reflect pharmacodynamic effects and treatment response of adalimumab in patients with axial spondyloarthritis—results from two randomized controlled trials

**DOI:** 10.1186/s13075-023-03132-5

**Published:** 2023-08-25

**Authors:** Helena Port, Signe Holm Nielsen, Peder Frederiksen, Sofie Falkenløve Madsen, Anne-Christine Bay-Jensen, Inge Juul Sørensen, Bente Jensen, Anne Gitte Loft, Ole Rintek Madsen, Mikkel Østergaard, Susanne Juhl Pedersen

**Affiliations:** 1 Department of Clinical Medicine, University of Copenhagen, Copenhagen, Denmark; 2 Nordic Bioscience A/S, Immunoscience, Herlev, Denmark; 3 Biomedicine and Biotechnology, Technical University of Denmark, Kongens Lyngby, Denmark; 4 Department of Biomedical Sciences, University of Copenhagen, Copenhagen, Denmark; 5 Department of Rheumatology and Spine Diseases, Righospitalet, Copenhagen, Denmark; 6 Department of Rheumatology, Aarhus University Hospital, Aarhus, Denmark; 7 Department of Clinical Medicine, University of Aarhus, Aarhus, Denmark; 8 Copenhagen Center for Arthritis Research, Center for Rheumatology and Spine Diseases, Rigshospitalet, Copenhagen, Denmark

**Keywords:** Axial spondyloarthritis, Extracellular matrix, Biomarkers, Adalimumab

## Abstract

**Objective:**

To investigate if extracellular matrix (ECM) blood-based biomarkers reflect the pharmacodynamic effect and response to TNF-α inhibitor therapy (adalimumab, ADA), in patients with axial spondyloarthritis (axSpA).

**Methods:**

We investigated ECM biomarkers in two randomized, double-blind, placebo-controlled trials of axSpA patients (DANISH and ASIM, *n* = 52 and *n* = 49, respectively) receiving ADA 40 mg or placebo every other week for 12 and 6 weeks, respectively, and thereafter ADA to week 48. Serum concentrations of degraded type I (C1M), II (C2M, T2CM), III (C3M), IV (C4M), VI (C6M), type X (C10C) collagen; metabolite of C-reactive protein (CRPM), prolargin (PROM), citrullinated vimentin (VICM), calprotectin (CPa9-HNE); and formation of type II (PRO‑C2), III (PRO‑C3), and VI (PRO‑C6) turnover of type IV collagen (PRO-C4) were measured at baseline and weeks 6 or 12, 24, and 48. The pharmacodynamic effect and treatment response to ADA was evaluated by linear mixed models, and correlations between biomarkers and clinical scores were assessed by Spearman’s correlation.

**Results:**

C1M, C3M, C4M, C6M, CRP, PRO-C4, and CPa9-HNE levels declined after 6 or 12 weeks in patients receiving ADA compared to placebo (all *p* < 0.05). Patients with AS Disease Activity Score C-reactive protein (ASDAS CRP) major improvement and/or clinically important improvement had significantly higher C1M, C3M, C4M, C6M, and PRO-C4 levels than patients with no/low improvement at baseline (all *p* < 0.05). Baseline levels of biomarkers showed weak to moderate correlations with ASDAS and structural damage scores.

**Conclusion:**

ECM metabolites showed a pharmacodynamic effect and were associated with ASDAS response during TNF-α inhibitor treatment in patients with axSpA.

**Supplementary Information:**

The online version contains supplementary material available at 10.1186/s13075-023-03132-5.

## Introduction

Axial spondyloarthritis (axSpA) is a chronic inflammatory disorder that predominantly affects the axial skeleton and the sacroiliac joints (SIJs) [1, 2]. Patients with axSpA are characterized by inflammation at the joints and entheses, destruction, and repair, as well as new bone formation [3]. The pathogenesis of axSpA is not clearly elucidated [4], but tumor necrosis factor α (TNF-α) plays an important role as a pro-inflammatory cytokine in the disease, resulting in joint inflammation [5]. Therefore, treatment with TNF-α inhibitors, such as adalimumab (ADA), is recommended after treatment failure to non-steroidal anti-inflammatory drugs (NSAIDs) [6]. TNF-α inhibitors have been shown to reduce disease activity within 12 weeks [7]. However, it may be detected much faster if assessed by blood-based biochemical markers, specifically pharmacodynamic biomarkers, which may assess the treatment response [8].

Tissue damage is a central pathological feature in axSpA [8], and several studies have shown that there is extracellular matrix (ECM) remodeling involved in the disease [9]. The ECM comprises a complex network of structural proteins and is essential for tissue function, structure, and homeostasis [10]. The main structural element of the ECM is collagens, and type I, II, III, IV, VI, and X collagen are the most abundant collagens of the joint tissue. During joint tissue turnover, the ECM is remodeled, generating fragments that are subsequently released into the circulation and can be quantified in blood using immunoassays to detect serological biomarkers [4, 8]. ECM biomarkers reflecting inflammation, bone, and connective tissue turnover have been associated with disease activity and progression when compared with clinical outcomes [11–13].

Blood-based biomarkers of metalloproteinases (MMP)-degraded type I (C1M), II (C2M), III (C3M), IV (C4M), and VI (C6M) collagen have shown higher levels in patients with ankylosing spondylitis (AS) compared to healthy controls [11, 14], and they have also presented elevated levels in patients with axSpA and psoriatic arthritis (PsA) compared to healthy [13, 15, 16]. Another MMP-degraded biomarker of type II collagen, T2CM, has reflected cartilage degradation in patients with osteoarthritis [17]. Prolargin and vimentin are found in connective tissue and cartilage and tendon, respectively [12, 18]. Degradation fragments of C-reactive protein (CRP) and prolargin (CRPM and PROM, respectively) have been associated with disease activity in axSpA and together with the citrullinated vimentin neoepitope biomarker, VICM, could separate AS patients from non-radiographic axSpA patients [12]. Biomarkers measuring type II, III, and VI collagen formation (PRO-C2, PRO-C3, and PRO-C6, respectively) have been found elevated in PsA compared to healthy individuals [16], and only PRO-C2 has also presented higher levels in axSpA [19]. PRO-C4, measuring ECM basement membrane turnover, has shown increased levels of axSpA compared to healthy controls [13]. Even though it has not previously been tested in axSpA, human neutrophil elastase (HNE)-mediated degradation of calprotectin (CPa9-HNE) measures neutrophil activity and has been highly associated with patients suffering from inflammatory bowel disease [20]. C6M, VICM, and PRO-C3 have also been associated with ASDAS (AS Disease Activity Score based on C-reactive protein) after TNF-α treatment [19]. Type X collagen degradation biomarkers (C10C) have shown lower levels in patients with axSpA in TNF-α treatment [21]. Nevertheless, only a few randomized placebo-controlled studies have investigated the association of biomarkers related to ECM turnover, such as MMP-3, tissue inhibitor of MMP (TIMP)-1, osteocalcin, type I collagen N-telopeptides (NTX), C-terminal of type II collagen neoepitope (C2C), and human cartilage glycoprotein-39 (YKL-40) [3, 22–25] and clinical measures of disease activity and treatment efficacy in patients with axSpA. Therefore, ECM biomarkers showing key features of axSpA should be further explored as potential pharmacodynamic biomarkers to aid in predicting and monitoring the treatment response of TNF-α inhibitors in patients with axSpA.

In the present study, we investigated a panel of 15 novel blood-based ECM biomarkers, reflecting MMP-driven inflammation and ECM tissue degradation (C1M, C2M, T2CM, C3M, C4M, C6M, C10C, CRPM, PROM, VICM), neutrophil activity (CPa9-HNE), fibrosis (PRO-C3 and PRO-C6), cartilage turnover (PRO-C2), and basement membrane turnover (PRO-C4) before and during treatment with the TNF-α inhibitor ADA based on two placebo-controlled axSpA studies. The aims were to investigate the potential of the ECM biomarkers to show the pharmacodynamic effect (i.e., changes during placebo vs active treatment) and to reflect treatment response to ADA based on the Bath AS Disease Activity Index (BASDAI) and ASDAS criteria.

## Methods

### Study design and population

**Fig. 1:**
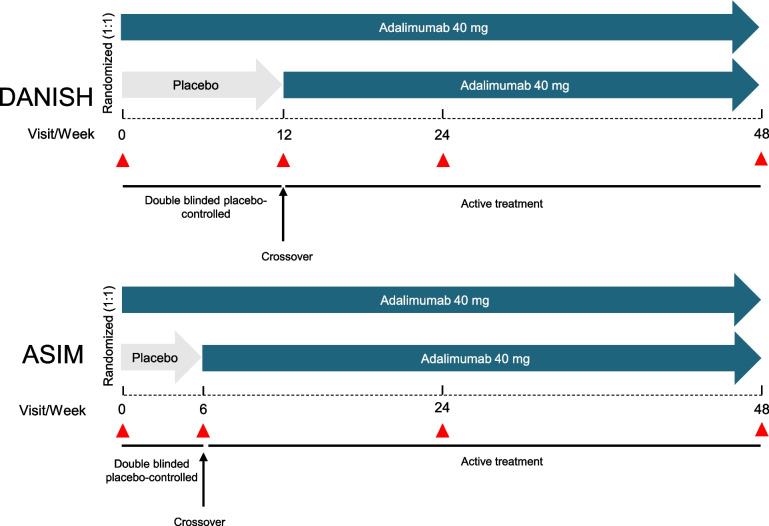
Study design for the DANISH and ASIM studies. Red triangles represent the visits where serum was collected. For the DANISH study, when analyzing the response to treatment, data of the placebo group at week 12 was considered week 0, and data from week 24 was considered week 12

Data from two randomized, double-blind, placebo-controlled investigator-initiated trials of axSpA patients were included in this study: the Danish Multicenter Study of Adalimumab in Spondyloarthritis (DANISH, NCT00477893) [26] and the Adalimumab in Axial Spondyloarthritis study (ASIM, NCT01029847) [27], see Fig. 1. The DANISH study included 52 patients with axSpA randomized 1:1 to receive subcutaneous (SC) injections of ADA 40 mg or placebo every other week from week 0 to week 12, followed by SC ADA 40 mg every other week to week 48. The ASIM study comprised 49 patients with axSpA randomized 1:1 to receive either SC ADA 40 mg or placebo every other week for 6 weeks, followed by SC ADA 40 mg every other week from week 6 to week 48. For both studies, the inclusion and exclusion criteria, study procedures, and main results for clinical outcomes and magnetic resonance imaging (MRI) have been described previously [26, 27].

Demographic and clinical data were acquired from all participants. In brief, the conventional patient-reported outcomes (PROs), clinical examinations, blood samples, and MRI scans of the SIJs and spine were performed at week 0 (before treatment initiation) for both studies and at weeks 6 (ASIM), 12 (DANISH), and 24 and 48 (DANISH and ASIM, respectively). Semi-coronal T1-weighted (T1W) and short tau inversion recovery (STIR) MRI sequences of the SIJs, and sagittal T1W and STIR sequences of the spine were evaluated according to the Spondyloarthritis Research Consortium of Canada (SPARCC) inflammation [28] and structural SIJ scores (SSS) for fat, erosion, backfill, and ankylosis [29]. MRI of the spine was evaluated according to the SPARCC spine inflammation [28] and Canada-Denmark (CanDen) Spine scores for inflammation, fat, erosion, and new bone formation [30, 31]. In addition, the Modified New York Criteria were applied on radiographs of the SIJs and the Modified Stoke AS Spine Score (mSASSS) on lateral lumbar and cervical spine radiographs.

### Biomarker assays

Serum samples were available from 49 and 45 patients in DANISH and ASIM, respectively. The samples had been stored at − 80 °C until biomarker analysis. A panel of ECM turnover biomarkers was measured in the serum using either manual solid-phase competitive enzyme-linked immunosorbent assay (ELISA) or the Immunodiagnostic Systems robotic platform (IDS-i10; Immunodiagnostic Systems (IDS), Bolden, Tyne & Wear, UK). The assessed biomarkers can be found in Table 1.

**Table 1:** The panel of biomarkers evaluated in this study

Biomarker	Description of the biomarker	Implication	Reference
C1M	MMP-2/9/13-degraded type I collagen	Interstitial matrix degradation	[32]
C2M	MMP (multiple)-degraded type II collagen	Cartilage degradation	[33]
T2CM	MMP-1/-13-degraded type II collagen	Cartilage degradation	[17]
C3M	MMP-9-degraded type III collagen	Interstitial matrix degradation	[34]
C4M	MMP (multiple)-degraded type IV collagen	Primarily basal lamina disruption	[35]
C6M	MMP-2/9-degraded type VI collagen	Microfibril degradation	[36]
C10C	Cathepsin-K-mediated degradation of type X collagen	Chondrocyte activity	[37]
PROM	MMP-1/13-cleaved prolargin	Interstitial matrix degradation	[18]
VICM	Citrullinated and MMP-degraded vimentin	Inflammation	[38]
CRP	C-reactive protein	Systemic inflammation	[26, 27]
CRPM	MMP-1/8-degraded C-reactive protein	Inflammation	[39]
PRO-C2	Type II collagen N-terminal pro-peptide	Cartilage formation	[40]
PRO-C3	Type II collagen N-terminal propeptide	Fibrosis	[41]
PRO-C4	Type IV 7S domain collagen	Basement membrane turnover	[42]
PRO-C6	Type VI collagen, alpha-3 chain, C5 domain	Fibrosis	[43]
CPa9-HNE	HNE-mediated degradation of calprotectin	Neutrophil activity and neutrophil extracellular trap formation, NETosis	[20]

*Abbreviations*: *MMP* Metalloproteinase, *HNE* Human elastase, *NET* Neutrophil extracellular traps

### Statistical analysis

Baseline characteristics of the patients are described as number (frequency) for categorical variables and as mean ± standard deviation (SD) for continuous variables. The Kruskal–Wallis test, Mann–Whitney test, or chi-square test were used when appropriate to examine the baseline differences between the groups.

To investigate the pharmacodynamic effect, the average percentage change in biomarker concentrations from baseline to week 12 or week 6 (DANISH and ASIM, respectively) between the placebo and ADA groups was evaluated by linear mixed models. The average percentage change was included as the dependent variable, group and visit as fixed effects, and patient-specific intercepts as random effects (to account for correlated measurements within patients).

To explore the treatment response, biomarker levels were compared between the groups defined by the BASDAI and ASDAS response criteria, where ASDAS was calculated based on CRP. A BASDAI response was defined as ≥ 50% reduction in the BASDAI index from baseline (BASDAI50 responder), and a BASDAI non-response was defined as < 50% reduction in BASDAI index from baseline (BASDAI50 non-responder). For ASDAS, we applied the cutoffs for treatment response defined by the Assessment of SpondyloArthritis International Society (ASAS): no/low improvement (NI) (ΔASDAS < 1.1), clinically important improvement (CII) (1.1 ≤ ΔASDAS < 2.0), and major improvement (MI) (ΔASDAS ≥ 2.0). Furthermore, another classification was done for ASDAS, where we pooled the groups who had at least a CII (≥ CII) (ΔASDAS ≥ 1.1, i.e., including CII and MI) as compared to NI (ΔASDAS < 1.1). To increase the number of patients when evaluating the response to treatment, in the DANISH study, data from the placebo group at week 24 (i.e., the visit after 12 weeks of active treatment with SC ADA) was pooled with the data from the ADA group at week 12 (i.e., for this group also the visit after 12 weeks of active treatment). In the ASIM study, the data were not pooled because the period where the patients received active treatment in both groups was different (i.e., at the visit at week 24, the placebo group had received 18 weeks of active treatment whereas the ADA group had received 24 weeks of active treatment).

The average level of biomarkers between response categories was evaluated by linear mixed models. Biomarker data was log-transformed and used as the dependent variable, with treatment response and visit included as fixed effects and patient-specific intercepts as random effects. For this analysis, estimates are shown back transformed, i.e., on the original scale of the markers. Contrast on the original scale represents the ratios of the geometric mean of serum levels for responders vs the geometric mean for the non-responders (BASDAI50 responder vs non-responder, ASDAS MI vs NI, ASDAS CI vs NI, and ASDAS ≥ CII vs NI).

To explore the correlations between serological biomarkers and clinical scores, Spearman’s correlations were calculated. Only correlations with a rho (*ρ*) value ≥ 0.3 or ≤  − 0.3 and a *p*-value < 0.01 were deemed important, as correlations below that threshold were considered very weak. Correlations between 0.30 and 0.39 were regarded as weak, 0.40 and 0.59 as moderate, and 0.60 and 0.79 as strong.

A significance level of 5% was used throughout the analyses, and false discovery rate correction for multiple testing was applied where indicated (targeting a false discovery rate of 5%). Data analyses were performed using R studio version 4.2.1 (R Foundation for Statistical Computing, Vienna, Austria, https://www.R-project.org; 2020). Graphical illustrations were created using GraphPad Prism version 9.00 for Windows (GraphPad Software, GraphPad Software, San Diego, CA, USA, www.graphpad.com).

## Results

### Baseline demographics

Baseline patient characteristics can be found in Table 2. In the DANISH and ASIM studies, 24 and 23 patients were randomized to receive ADA treatment and 25 and 22 patients to placebo, respectively.

**Table 2:** Baseline clinical characteristics of the patients in the DANISH and ASIM studies*

Demographic feature	DANISH			ASIM			DANISH vs ASIM
	**Total (*N* = 49)**	**Adalimumab (*N* = 24)**	**Placebo (*N* = 25)**	**Total (*N* = 45)**	**Adalimumab (*N* = 22)**	**Placebo (*N* = 23)**	***p*-value**
Age, years	39.5 (11.2)	40.9 (12.5)	38.2 (9.9)	37.6 (9.9)	39.8 (11.4)	35.4 (7.9)	0.47
Sex, male	38 (77.6%)	18 (75.0%)	20 (80.0%)	25 (55.6%)	13 (59.1%)	12 (52.2%)	0.02
HLA–B27 positive	43 (87.8%)	23 (95.8%)	20 (80.0%)	34 (75.6%)	16 (72.7%)	18 (78.3%)	0.12
Symptom duration, years	10.9 (8.2)	12.1 (7.1)	9.9 (9.1)	12.4 (11.2)	14.5 (14.1)	10.3 (7.3)	0.98
ASDAS	3.2 (0.8)	3.3 (0.8)	3.2 (0.8)	3.5 (0.8)	3.6 (0.7)	3.5 (0.9)	0.1
BASDAI	6 (1.7)	6 (1.4)	6.1 (1.9)	6.4 (1.4)	6.3 (1.2)	6.4 (1.6)	< 0.01
BASFI	4.6 (1.9)	4.8 (1.7)	4.3 (2.1)	5.1 (2.1)	5.2 (2.0)	5.0 (2.2)	0.22
BASMI	3.3 (2.0)	3.3 (2.0)	3.3 (2.1)	2.7 (2.0)	2.6 (2.0)	2.7 (2.0)	0.13
Fulfillment of the modNY criteria	46 (93.9%)	24 (100.0%)	22 (88.0%)	27 (60.0%)	13 (59.1%)	14 (60.9%)	< 0.01
SPARCC inflammation score	8.2 (10.4)	5.0 (6.6)	11.2 (12.5)	7.4 (8.5)	5.8 (5.9)	9.1 (10.4)	0.74
SPARCC SSS fat score	15.6 (15.4)	18.7 (16.5)	12.6 (13.9)	6.4 (8.0)	6.4 (7.5)	6.4 (8.7)	< 0.01
SPARCC SSS erosion score	2.5 (4.1)	1.3 (2.1)	3.7 (5.2)	3.0 (3.2)	2.4 (2.7)	3.5 (3.7)	0.24
SPARCC SSS backfill score	5.7 (5.6)	6.5 (6.4)	4.9 (4.7)	0.8 (1.7)	0.9 (1.9)	0.8 (1.4)	< 0.01
SPARCC spine inflammation score	13.6 (16.5)	15.9 (19.0)	11.4 (13.8)	8.2 (11.3)	9.5 (12.6)	6.8 (10.1)	0.11
CanDen spine inflammation score	16.0 (21.2)	15.6 (15.1)	16.3 (26.0)	6.7 (9.0)	8.1 (10.4)	5.3 (7.5)	0.01
CanDen spine erosion score	0.1 (0.5)	0.2 (0.7)	0.0 (0.2)	0.5 (0.9)	0.4 (0.7)	0.6 (1.0)	< 0.01
CanDen Spine fat score	19.1 (24.7)	28.6 (31.2)	10.1 (10.7)	5.2 (9.2)	7.8 (11.9)	2.7 (4.8)	< 0.01
CanDen Spine new bone formation score​	12.8 (33.0)	21.6 (45.1)	4.4 (8.7)	10.4 (22.5)	13.2 (28.1)	7.8 (15.7)	0.61
mSASSS	8.6 (13.2)	11.1 (15.9)	6.1 (9.5)	7.8 (14.0)	9.1 (15.5)	6.5 (12.6)	0.72
C1M, ng/mL	103.2 (78.4)	109.9 (73.1)	96.8 (84.2)	77.8 (76.6)	81.1 (61.6)	74.6 (90.0)	0.02
C3M, ng/mL	13.9 (3.0)	14.2 (3.1)	13.7 (3.1)	13.4 (2.6)	13.7 (2.5)	13.1 (2.6)	0.51
C4M, ng/mL	33.6 (8.6)	33.7 (9.4)	33.5 (7.8)	32.4 (7.4)	33.6 (7.2)	31.3 (7.6)	0.64
C6M, ng/mL	22.7 (7.8)	22.9 (7.1)	22.5 (8.6)	21.3 (6.6)	22.3 (7.1)	20.4 (6.0)	0.37
CRP, mg/L	16.6 (23.5)	16.9 (18.4)	16.2 (28.0)	10.3 (14.6)	11.3 (12.3)	9.3 (16.7)	0.02
CRPM, ng/mL	11.8 (4.1)	11.5 (2.8)	12.1 (5.1)	13.8 (14.9)	15.4 (20.7)	12.2 (5.3)	0.67
PROM, ng/mL	0.2 (0.1)	0.2 (0.1)	0.2 (0.1)	0.2 (0.1)	0.2 (0.1)	0.2 (0.1)	0.6
VICM, ng/mL	6.8 (5.3)	7.6 (6.1)	6.1 (4.5)	3.6 (2.7)	4.4 (3.3)	2.9 (1.8)	< 0.01
CPa9-HNE, ng/mL	80.9 (33.5)	87.0 (36.5)	75.1 (29.9)	128.3 (61.4)	135.0 (57.3)	121.9 (65.7)	< 0.01
C2M, ng/mL	20.3 (3.4)	20.3 (2.6)	20.4 (4.1)	21.7 (5.3)	21.8 (4.9)	21.6 (5.8)	0.28
T2CM, ng/mL	6.3 (2.1)	6.2 (1.8)	6.4 (2.4)	5.4 (1.0)	5.4 (0.9)	5.4 (1.1)	< 0.01
C10C, ng/mL	2634.9 (555.2)	2755.6 (462.6)	2519.0 (618.8)	2585.0 (655.6)	2655.3 (811.9)	2517.8 (470.0)	0.37
PRO-C3, ng/mL	11.0 (2.1)	10.9 (2.0)	11.1 (2.2)	10.5 (2.8)	10.3 (2.1)	10.6 (3.3)	0.08
PRO-C4, ng/mL	7026.4 (832.7)	6998.8 (501.6)	7052.9 (1069.6)	6592.2 (775.7)	6552.8 (822.0)	6629.9 (745.2)	0.04
PRO-C6, ng/mL	8.2 (2.6)	7.9 (2.4)	8.5 (2.9)	7.4 (1.7)	7.5 (1.9)	7.4 (1.6)	0.31
PRO-C2 ng/mL	21.9 (8.4)	22.3 (9.6)	21.6 (7.2)	18.7 (14.3)	16.6 (13.0)	20.8 (15.4)	< 0.01

*Except where indicated otherwise, mean ± SD is presented. Study differences were analyzed by Mann-Whitney tests or chi-square test, as appropriate. *Abbreviations*: *ASDAS* Assess Disease Activity in Ankylosing Spondylitis, *BASDAI* Bath Ankylosing Spondylitis Disease Activity Index (scale 0–10), *BASFI* Bath Ankylosing Spondylitis Functional Index (scale 0–10), *BASMI* Bath Ankylosing Spondylitis Metrology Index (scale 0–10), *modNY criteria* Modified New York Criteria, *SPARCC* Spondyloarthritis Research Consortium of Canada, *SSS* SI joint structural lesion score, *CanDen* Canada and Denmark, *mSASSS* Modified Stoke Ankylosing Spondylitis Score (scale 0–40), *C1M* metalloproteinase (MMP)-2/9/13-degraded type I collagen, *C3M* MMP-degraded type III collagen, *C4M* MMP (multiple)-degraded type IV collagen, *C6M* MMP-2/9-degraded type VI collagen, *CRP* C-reactive protein, *CRPM* CRP metabolite, *PROM* MMP-1 and MMP-13-mediated degradation of prolargin, *VICM* citrullinated and MMP-degraded vimentin, *CPa9-HNE* human elastase (HNE)-mediated degradation of calprotectin, *C2M* MMP (multiple)-degraded type II collagen, *T2CM* MMP-1/13-mediated degradation of type II collagen, *C10C* cathepsin-K-mediated degradation of type X collagen *PRO-C3* pro-peptide of type III collagen, *PRO-C4* type IV 7S domain collagen, *PRO-C6* type VI alpha-3 chain collagen, *PRO-C2* pro-peptide of type II collagen

When comparing the two studies, patients enrolled in the DANISH study had a higher fraction of male participants, mean BASDAI, percent of the fulfillment of the Modified New York Criteria, CanDen spine inflammation score, CanDen spine erosion score, and CanDen spine fat compared to the ASIM study (Table 2, all *p*-value < 0.02).

Baseline characteristics of the patients based on the three classifications of response to ADA can be found in the supplementary material (Additional file 1: Table S1, Table S2, and Table S3).

### Pharmacodynamic effects of ADA treatment on serum biomarkers of ECM degradation and inflammation

**Fig. 2:**
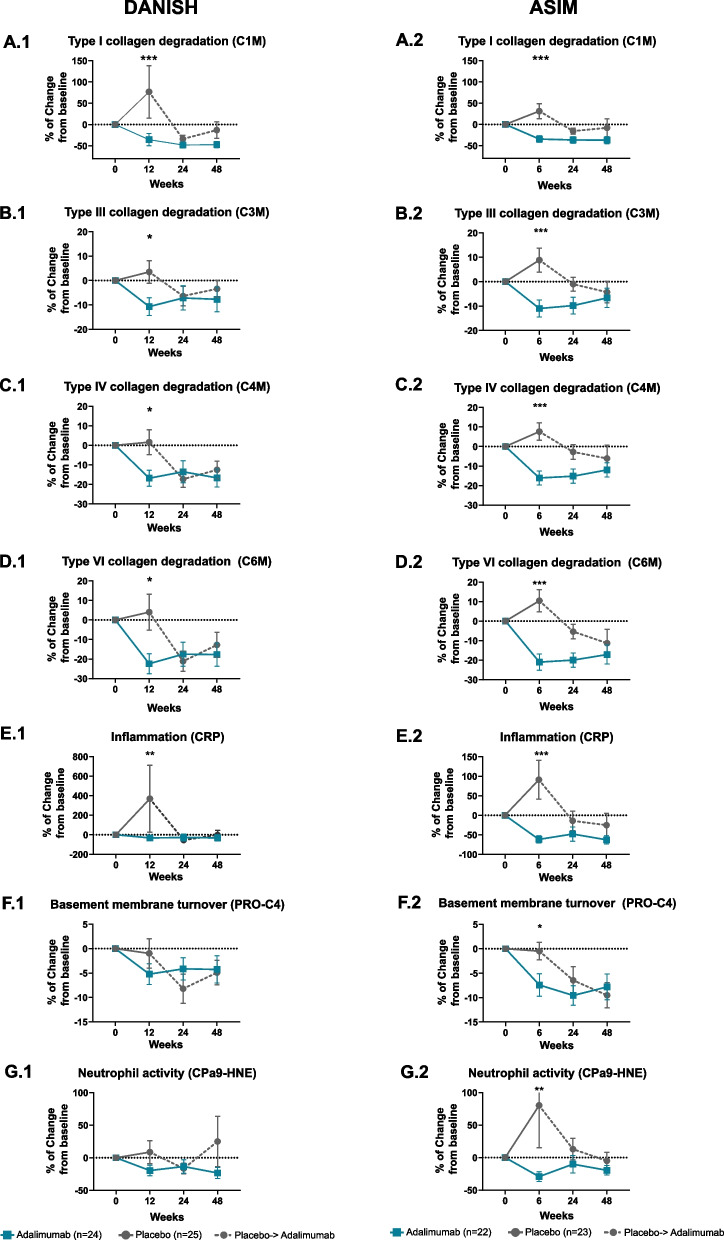
The pharmacodynamic effect of ADA treatment in the DANISH and ASIM studies. Percentage (%) change from baseline of the placebo group vs the ADA group of the selected ECM biomarkers. **A.1**–**G.1** DANISH study. **A.2**–**G.2** ASIM study. *p*-values at weeks 12 and 6 were adjusted for multiple comparisons by the false rate discovery method, and a significant difference is displayed by the following asterisks: *p < 0.05, **p < 0.01, ***p < 0.001. Dashed points determine the crossover from placebo to active ADA treatment. Data are presented as mean ± standard error of the mean. Only biomarkers that obtained significance in the analyses are shown here; the rest are provided in Additional file 1: Fig. S1. *Abbreviations*: C1M, metalloproteinase (MMP)-2/9/13-degraded type I collagen; C3M, MMP-degraded type III collagen; C4M, MMP (multiple)-degraded type IV collagen; C6M, MMP-2/9-degraded type VI collagen; PRO-C4, type IV 7S domain collagen; CPa9-HNE, HNE-mediated degradation of calprotectin

In the DANISH study, the percentage of change from baseline to week 12 of type I (C1M), type III (C3M), type IV (C4M), and type VI (C6M) collagen degradation and CRP was significantly different between the ADA-treated group and the placebo group (*p* < 0.001, *p* < 0.05, *p* < 0.05, *p* < 0.05, *p* < 0.01, respectively; Fig. 2A.1–E.1). For all these biomarkers, the percentage of change was negative in the ADA group, reflecting a decline in the biomarker levels. In the ASIM study, the percentage of change from baseline to week 6 of type I (C1M), type III (C3M), type IV (C4M), and type VI (C6M) collagen degradation; basement membrane turnover (PRO-C4); neutrophil activity marker (CPa9-HNE); and CRP was significantly different between the ADA-treated group and the placebo group (all *p* < 0.001, Fig. 2.A.2–G.2). For all these biomarkers, the percentage of change was negative in the ADA group, showing a decrease in the biomarker levels. Furthermore, when the placebo group of both studies started receiving ADA treatment (i.e., after the crossover), the levels of the biomarkers also showed a negative percentage of change, and most of the biomarkers approached the levels of the ADA-treated group during the observation time (Fig. 2).

The remaining investigated biomarkers, CRPM, PROM, VICM, C2M, T2CM, C10C, PRO-C3, PRO-C6, and PRO-C2, did not show any significant percentage of change between the ADA and placebo groups in either DANISH or ASIM (Additional file 1: Fig. S1).

### Serum ECM biomarkers of inflammation and degradation in BASDAI and ASDAS responders vs non-responders

In the DANISH study, we observed that BASDAI50 responders and patients who achieved ASDAS CII and ASDAS MI tended to have higher biomarker levels at baseline compared to BASDAI non-responders and patients with ASDAS NI, respectively. Specifically, patients with ASDAS MI had significantly higher levels of type I (C1M), III (C3M), IV (C4M), and VI (C6M) collagen degradation and CRP at baseline compared to patients with ASDAS NI (*p* < 0.001, *p* < 0.05, *p* < 0.001, *p* < 0.01, and *p* < 0.001, Table 3).

**Table 3:** Comparison of biomarker levels based on a 50% reduction in the BASDAI index and ASDAS criteria from baseline to week 12 or week 24 in the DANISH and ASIM studies, respectively

Biomarker	Baseline								After treatment^a^							
	**DANISH**				**ASIM**				**DANISH**				**ASIM**			
	**BASDAI R vs NR**	**ASDAS CII vs NI**	**ASDAS MI vs NI**	**ASDAS ≥ CII vs NI**	**BASDAI R vs NR**	**ASDAS CII vs NI**	**ASDAS MI vs NI**	**ASDAS ≥ CII vs NI**	**BASDAI R vs NR**	**ASDAS CII vs NI**	**ASDAS MI vs NI**	**ASDAS ≥ CII vs NI**	**BASDAI R vs NR**	**ASDAS CII vs NI**	**ASDAS MI vs NI**	**ASDAS ≥ CII vs NI**
C1M	1.43 [0.99, 2.07]	1.43 [0.94, 2.18]	**2.79 [1.85, 4.19]**^**§**^	**2.04 [1.39, 3.0]**^**‡**^	1.46 [0.93, 2.3]	1.18 [0.71, 2.0]	1.90 [1.19, 3.0]	1.58 [1.03, 2.4]	0.79 [0.54, 1.16]	0.77 [0.51, 1.18]	0.79 [0.52, 1.19]	0.78 [0.53, 1.1]	0.88 [0.56, 1.4]	0.92 [0.55, 1.5]	0.94 [0.59, 1.5]	0.93 [0.60, 1.4]
C3M	1.07 [0.94, 1.21]	1.13 [0.97, 1.32]	**1.29 [1.11, 1.50]**^**†**^	**1.21 [1.06, 1.4]**^**†**^	1.04 [0.89, 1.2]	0.99 [0.83, 1.2]	1.06 [0.91, 1.2]	1.03 [0.89, 1.2]	0.92 [0.81, 1.04]	1.02 [0.87, 1.19]	0.96 [0.82, 1.11]	0.99 [0.86, 1.1]	0.97 [0.83, 1.1]	1.05 [0.88, 1.3]	0.93 [0.80, 1.1]	0.98 [0.84, 1.1]
C4M	1.14 [0.99, 1.32]	1.14 [0.97, 1.35]	**1.47 [1.25, 1.72]**^**§**^	**1.31 [1.13, 1.5]**^**‡**^	1.04 [0.88, 1.2]	0.99 [0.81, 1.2]	1.11 [0.93, 1.3]	1.06 [0.90, 1.2]	0.94 [0.81, 1.08]	0.91 [0.77, 1.07]	0.98 [0.84, 1.15]	0.95 [0.82, 1.1]	0.90 [0.76, 1.1]	0.98 [0.80, 1.2]	0.90 [0.75, 1.1]	0.93 [0.79, 1.1]
C6M	1.09 [0.91, 1.30]	1.21 [0.98, 1.49]	**1.54 [1.25, 1.89]**^**‡**^	**1.37 [1.14, 1.7]**^**‡**^	1.27 [1.03, 1.6]	1.25 [0.99, 1.6]	1.24 [1.00, 1.5]	1.24 [1.02, 1.5]	0.90 [0.75, 1.08]	1.00 [0.81, 1.24]	1.00 [0.82, 1.23]	1.00 [0.83, 1.2]	1.04 [0.84, 1.3]	1.14 [0.89, 1.4]	0.99 [0.79, 1.2]	1.04 [0.85, 1.3]
CRP	1.83 [0.99, 3.38]	1.48 [0.76, 2.89]	**6.83 [3.58,13.06]**^**§**^	**3.35 [1.77, 6.3]**^**‡**^	2.50 [0.84, 7.4]	1.33 [0.37, 4.7]	3.65 [1.16,11.5]	2.47 [0.86, 7.1]	0.70 [0.37, 1.31]	0.78 [0.40, 1.52]	0.89 [0.46, 1.69]	0.83 [0.44, 1.6]	0.71 [0.24, 2.1]	0.95 [0.27, 3.4]	0.78 [0.25, 2.5]	0.85 [0.29, 2.4]
CRPM	1.22 [1.01, 1.48]	1.00 [0.77, 1.28]	1.30 [1.02, 1.66]	1.15 [0.92, 1.4]	1.16 [0.87, 1.5]	1.03 [0.73, 1.4]	1.10 [0.81, 1.5]	1.07 [0.81, 1.4]	0.99 [0.82, 1.20]	0.92 [0.72, 1.18]	0.95 [0.75, 1.22]	0.94 [0.75, 1.2]	0.94 [0.70, 1.3]	0.94 [0.67, 1.3]	0.96 [0.70, 1.3]	0.95 [0.72, 1.3]
PROM	0.80 [0.63, 1.03]	1.08 [0.78, 1.49]	0.88 [0.65, 1.21]	0.97 [0.73, 1.3]	1.08 [0.84, 1.4]	1.13 [0.84, 1.5]	1.35 [1.03, 1.8]	1.26 [0.98, 1.6]	0.75 [0.58, 0.96]	1.01 [0.73, 1.40]	0.78 [0.57, 1.07]	0.88 [0.67, 1.2]	0.97 [0.75, 1.3]	1.05 [0.78, 1.4]	1.19 [0.91, 1.6]	1.14 [0.89, 1.5]
VICM	1.28 [0.85, 1.92]	1.04 [0.61, 1.76]	1.82 [1.10, 3.01]	1.41 [0.90, 2.2]	1.22 [0.82, 1.8]	1.30 [0.83, 2.0]	1.16 [0.77, 1.7]	1.21 [0.83, 1.8]	0.95 [0.62, 1.45]	0.74 [0.43, 1.25]	0.93 [0.56, 1.53]	0.84 [0.53, 1.3]	1.06 [0.71, 1.6]	1.38 [0.88, 2.2]	0.93 [0.61, 1.4]	1.08 [0.73, 1.6]
CPa9-HNE	1.20 [0.81, 1.79]	0.72 [0.46, 1.12]	1.28 [0.83, 1.96]	0.98 [0.66, 1.5]	1.43 [0.96, 2.2]	1.36 [0.85, 2.2]	1.38 [0.90, 2.1]	1.38 [0.93, 2.0]	1.02 [0.68, 1.53]	0.59 [0.38, 0.92]	0.79 [0.51, 1.21]	0.69 [0.47, 1.0]	1.05 [0.69, 1.6]	1.14 [0.70, 1.8]	0.97 [0.63, 1.5]	1.03 [0.69, 1.5]
C2M	1.07 [0.83, 1.37]	0.89 [0.63, 1.24]	1.13 [0.82, 1.56]	1.01 [0.76, 1.3]	1.14 [0.97, 1.3]	1.16 [0.96, 1.4]	1.12 [0.94, 1.3]	1.14 [0.97, 1.3]	0.85 [0.66, 1.10]	0.80 [0.57, 1.12]	0.84 [0.61, 1.16]	0.82 [0.62, 1.1]	1.07 [0.90, 1.3]	1.11 [0.91, 1.4]	1.04 [0.87, 1.2]	1.07 [0.90, 1.3]
T2CM	0.88 [0.72, 1.07]	1.05 [0.80, 1.37]	0.93 [0.72, 1.21]	0.98 [0.78, 1.2]	0.99 [0.85, 1.1]	0.99 [0.83, 1.2]	1.00 [0.85, 1.2]	1.00 [0.86, 1.2]	0.87 [0.71, 1.06]	1.06 [0.81, 1.38]	0.85 [0.66, 1.10]	0.94 [0.75, 1.2]	0.99 [0.85, 1.2]	1.04 [0.87, 1.2]	0.96 [0.81, 1.1]	0.99 [0.85, 1.1]
C10C	1.06 [0.94, 1.18]	0.98 [0.84, 1.13]	1.02 [0.88, 1.17]	1.00 [0.88, 1.1]	1.03 [0.87, 1.2]	1.14 [0.93, 1.4]	1.08 [0.89, 1.3]	1.10 [0.93, 1.3]	1.05 [0.94, 1.18]	1.04 [0.90, 1.20]	1.08 [0.94, 1.24]	1.06 [0.94, 1.2]	0.99 [0.83, 1.2]	1.04 [0.84, 1.3]	1.02 [0.85, 1.2]	1.03 [0.87, 1.2]
PRO-C3	0.97 [0.86, 1.09]	1.11 [0.97, 1.27]	1.02 [0.90, 1.17]	1.06 [0.94, 1.2]	0.86 [0.75, 1.0]	0.91 [0.76, 1.1]	0.87 [0.74, 1.0]	0.88 [0.76, 1.0]	0.98 [0.87, 1.11]	1.03 [0.89, 1.18]	1.03 [0.90, 1.18]	1.03 [0.92, 1.2]	0.89 [0.76, 1.0]	0.93 [0.78, 1.1]	0.91 [0.78, 1.1]	0.92 [0.79, 1.1]
PRO-C4	1.02 [0.94, 1.09]	1.08 [0.98, 1.19]	1.10 [1.00, 1.21]	**1.09 [1.00, 1.2]**	1.12 [1.02, 1.2]	1.10 [0.99, 1.2]	1.14 [1.03, 1.3]	1.13 [1.03, 1.2]^**†**^	0.97 [0.90, 1.05]	1.03 [0.93, 1.14]	1.01 [0.92, 1.12]	1.02 [0.94, 1.1]	1.00 [0.91, 1.1]	1.02 [0.92, 1.1]	1.01 [0.91, 1.1]	1.01 [0.93, 1.1]
PRO-C6	0.93 [0.80, 1.09]	0.97 [0.78, 1.20]	1.01 [0.82, 1.24]	0.99 [0.83, 1.2]	0.95 [0.82, 1.1]	1.02 [0.85, 1.2]	1.02 [0.86, 1.2]	1.02 [0.88, 1.2]	0.90 [0.77, 1.05]	0.97 [0.78, 1.20]	0.96 [0.78, 1.18]	0.96 [0.80, 1.2]	0.90 [0.77, 1.0]	0.93 [0.78, 1.1]	0.95 [0.81, 1.1]	0.95 [0.81, 1.1]
PRO-C2	0.91 [0.72, 1.14]	1.09 [0.80, 1.49]	0.98 [0.72, 1.32]	1.03 [0.79, 1.3]	1.17 [0.79, 1.7]	1.02 [0.65, 1.6]	1.21 [0.80, 1.8]	1.13 [0.78, 1.7]	0.91 [0.72, 1.14]	0.99 [0.73, 1.36]	1.00 [0.74, 1.36]	1.00 [0.77, 1.3]	1.16 [0.79, 1.7]	0.98 [0.62, 1.5]	1.25 [0.83, 1.9]	1.14 [0.78, 1.7]

Data are presented as the ratio of average serum biomarker levels with 95% CI between the groups. *p*-values were adjusted for multiple comparisons by the false rate discovery method, and significance is shown as ^†^*p* < 0.05, ^‡^*p* < 0.01, and ^§^*p* < 0.001

*Abbreviations*: *BASDAI* Bath Ankylosing Spondylitis Disease Activity Index, *R* responder, *NR* non-responder, *ASDAS* Assess Disease Activity in Ankylosing Spondylitis, *MI* major improvement, *CII* clinically important improvement, *NI* no improvement, *C1M* metalloproteinase (MMP)-2/9/13-degraded type I collagen, *C3M* MMP-degraded type III collagen, *C4M* MMP (multiple)-degraded type IV collagen, *C6M* MMP-2/9-degraded type VI collagen, *CRP* C-reactive protein, *CRPM* C-reactive protein metabolite, *PROM* MMP-1- and MMP-13-mediated degradation of prolargin, *VICM* citrullinated and MMP-degraded vimentin, *CPa9-HNE* HNE-mediated degradation of calprotectin, *C2M* MMP (multiple)-degraded type II collagen, *T2CM* MMP-1/13-mediated degradation of type II collagen, *C10C* cathepsin-K-mediated degradation of type X collagen, *PRO-C3* pro-peptide of type III collagen, *PRO-C4* type IV 7S domain collagen, *PRO-C6* type VI alpha-3 chain collagen, *PRO-C2* pro-peptide of type II collagen

^a^Response to treatment was considered after 12 or 24 weeks of treatment (DANISH and ASIM, respectively). In the DANISH study, data from the placebo group at week 12 was considered baseline, and data from week 24 was considered as week 12 of treatment

Furthermore, patients achieving at least an ASDAS clinically important (≥ CII) also had significantly higher levels of type I (C1M), III (C3M), IV (C4M), and VI (C6M) collagen degradation and CRP compared to ASDAS NI at baseline (all *p* < 0.05, Table 3). In the ASIM study, we observed that BASDAI responders, patients with ASDAS CII, ASDAS MI, and patients with an ASDAS ≥ CII tended to have numerically higher biomarker levels at baseline compared to BASDAI50 non-responders or patients with ASDAS NI, respectively. Only PRO-C4 reached statistical significance in the group of patients with an ASDAS ≥ CII as compared to ASDAS NI (*p* < 0.05, Table 3).

After 12 weeks of treatment in the DANISH study, the biomarker levels showed a numerical trend to be lower or equal in BASDAI50 responders vs non-responders, in patients with ASDAS ≥ CII, ASDAS CII, or ASDAS MI vs ASDAS NI, respectively. After 24 weeks of treatment in the ASIM study, biomarker levels also tended to be lower or equal in BASDAI50 responders vs non-responders, patients with an ASDAS ≥ CII, ASDAS CII, or ASDAS MI vs ASDAS NI, respectively, but none reached statistical significance.

### ECM remodeling biomarkers’ correlation with clinical assessment of disease activity or severity of axSpA

We further examined the correlations between circulating ECM metabolites and clinical parameters. In the DANISH study, moderate correlations were observed between ASDAS and the biomarkers C1M C3M, C4M, C6M, VICM, and CPa9-HNE (*ρ* = 0.68 [0.47, 0.82], 0.44 [0.15, 0.66], 0.53 [0.27, 0.72], 0.63 [0.40, 0.79], 0.22 [− 0.09, 0.50], and 0.64 [0.42, 0.79]) and a weak correlation between mSASSS and PROM (*ρ* = 0.33 [0.02, 0.58], Additional file 1: Table S4). In the ASIM study, weak correlations were observed between age and CPa9-HNE (*ρ* =  − 0.34 [− 0.58, − 0.05]). Moderate to strong correlations were observed between ASDAS and C1M, C3M, C4M, C6M, CPa9-HNE, and PRO-C4 (*ρ* = 0.70 [0.51, 0.83], 0.36 [0.07, 0.59], 0.51 [0.25, 0.70], 0.48 [0.21, 0.68], and 0.51 [0.25, 0.70], Additional file 1: Table S5). Weak correlations were found between SPARCC inflammation score and PRO-C6 (*ρ* = 0.31 [0.01, 0.55]), SPARCC SSS fat score, and C10C (*ρ* =  − 0.31 [− 0.55, − 0.01]). Moderate and weak correlations were observed between SPARCC SSS erosion score and CRPM and PRO-C6, respectively (*ρ* = 0.44 [0.17, 0.65] and 0.36 [0.07, 0.60]). SPARCC SSS ankylosis score showed a moderate correlation with PRO-C6 (*ρ* =  − 0.43 [− 0.65, − 0.16]) while mSASSS correlated with PRO-C3 (*ρ* =  − 0.31 [− 0.56, − 0.02], Additional file 1: Table S5).

## Discussion

In this study, we evaluate the capacity of ECM biomarkers to reflect the pharmacodynamic effects and response to the TNF-α inhibitor ADA in two axSpA randomized double-blind placebo-controlled studies. To our knowledge, this is the first study where an extensive panel of ECM serological biomarkers has been measured in a randomized controlled study of the effect of a TNF-α inhibitor in patients with axSpA. The main findings were as follows: (i) C1M, C3M, C4M, C6M, and CRP were decreased over time in patients receiving ADA compared to the placebo group; (ii) C1M, C3M, C4M, and C6M could discriminate patients with ASDAS MI and patients achieving at least an ASDAS ≥ CII vs ASDAS NI at baseline; (iii) PRO-C4 could discriminate between patients with an ASDAS ≥ CII and patients with ASDAS NI at baseline; and (iv) mild-moderate correlations were observed between ECM biomarkers and clinical scores.

Determining the optimal treatment for axSpA in a timely and non-invasive manner remains a challenge in clinical practice [44]. Blood-based biomarkers might ameliorate this unmet need as they are non-invasive, more accessible, and less expensive tools compared to other available resources. MRI is a useful tool for evaluating the status and change in axial inflammation before and during TNF-α inhibitor therapy, but it is unclear how early the benefit can be detected [9]. Furthermore, MRI techniques are costly and require experienced professionals to interpret the findings. The early identification of patients who are most likely to exhibit improvement after TNF-α inhibitors using biomarkers might assist clinicians in their treatment decisions.

In this study, we also demonstrated the discriminant capacity and sensitivity to change of ECM biomarkers when patients are treated with ADA, representing an effective treatment in this patient group of two well-characterized studies, DANISH and ASIM, the former presenting patients with higher disease activity. It is well known that axSpA is characterized by inflammation [45]. Yet the nature of the inflammation is not fully elucidated, changes in the ECM, specifically collagen turnover, have been associated with inflammation in patients with axSpA [9, 13, 19]. We found that biomarkers related to MMP-driven inflammation and ECM tissue degradation, C1M, C3M, C4M, and C6M, reflected the pharmacodynamic effect of the TNF-α inhibitor ADA. In agreement with our findings, Holm Nielsen et al. [19] assessed C3M, C4M, C6M, and VICM in patients with axSpA treated with TNF-α inhibitor and observed that C6M and VICM were significantly decreased after two weeks of treatment. Schett et al. [16] found reduced levels of C1M, C3M, C4M, and C6M in patients with PsA after treatment with guselkumab, targeting the interleukin 23 p19 subunit, which is involved in the inflammation processes in SpA. However, Visvanathan et al. [46] observed that a C-terminal cross-linking telopeptide of type I collagen marker (CTX-I) did not show any differences between infliximab (TNF-α inhibitor) and placebo-treated patients with axSpA. On the other hand, collagen formation biomarkers, PRO-C3 and PRO-C6, were not altered in response to ADA, in agreement with Holm Nielsen et al. [19], suggesting that it may mainly suppress inflammation-driven tissue degradation and not the development of fibrosis. The latter is also in agreement with MRI studies, including the DANISH study, showing progression in structural damage during the first year after initiation of TNF-α inhibitor [26, 47, 48]. We did not detect any changes in either type II collagen degradation or formation, C2M and PRO-C2, respectively. These results are similar to those reported by Kim et al. [49], who observed no differences in serum biomarkers of type II collagen formation as a result of infliximab therapy in patients with axSpA. In contrast, it has been found that a type II collagen degradation fragment level (C2C) was reduced in etanercept-treated patients compared to placebo-treated patients [25], and a urinary type II collagen C-telopeptide marker (CTX-II) was suppressed after ADA treatment [24]. These results might implicate that C2M could not evaluate the cartilage turnover in this study. In only one of the two studies investigated, we observed that PRO-C4, a basement membrane turnover marker, and CPa9-HNE, a neutrophil activity marker, were also decreased after ADA treatment, which suggests that ECM turnover and immune-related activity might also be inhibited after TNF-α inhibitor treatment. The additional assessed biomarkers did not show any changes between ADA and placebo in the pharmacodynamic effect of ADA.

Additionally, we investigated the discriminative ability of the ECM biomarkers to differentiate clinical responders vs non-responders to ADA based on the BASDAI50 and ASDAS response criteria. From our knowledge, this is the first study that investigates the association of an extensive ECM biomarker panel with the ASDAS criteria in a randomized placebo-controlled study design. No significant differences were observed between BASDAI50 responders and non-responders at any time point. However, we found that patients achieving at least an ASDAS change > CII had higher levels of C1M, C3M, C4M, C6M, CRP, and PRO-C4 at baseline. These results emphasize the association of these biomarkers with clinical response. Specifically, increased inflammation-driven ECM degradation, reflected by these biomarkers, may lead to a better response to ADA treatment in patients with axSpA when assessed by the ASDAS criteria. On the other hand, no differences were observed in type II, III, and IV collagen formation markers in patients fulfilling vs not fulfilling neither the BASDAI nor ASDAS response criteria. Specifically for type III collagen formation, Holm Nielsen et al. [19] observed that patients with no improvement in ASDAS (after 22 weeks of TNF-α inhibitor treatment) had higher baseline levels of PRO-C3 than patients with CI and MI in ASDAS. Further research in this area could contribute to the development of personalized treatment strategies for axSpA patients, considering their unique disease characteristics and ECM remodeling patterns.

As a complementary analysis, we assessed the correlations between the biomarker data and the clinical scores. We observed weak-moderate correlations between ASDAS and C1M, C3M, C4M, C6M, VICM, and CPa9-HNE. In a previous work [13], we found mild-moderate correlations with the SPARCC SSS ankylosis score and C3M, C4M, C6M, and PROM. However, in this study, we observed significant weak-moderate correlations between SPARCC SSS (inflammation, fat, and erosion) and C10C, CRPM, and PRO-C6. Further studies are therefore needed to clarify the relation between the biomarkers and structural damage in patients with axSpA.

The strength of this study was the extensive panel of ECM biomarkers to evaluate the ADA effect in two independent well-characterized axSpA cohorts. The results of this study emphasize the great potential of ECM biomarkers for the evaluation of pharmacodynamic effects in randomized placebo-controlled studies, even in studies with small sample sizes (< 50) (Table 2). Moreover, in subsequent studies, by combining information from ECM remodeling biomarkers with other clinical scores, clinicians may potentially obtain a more comprehensive picture of the patient’s disease status and response to treatment.

There are several limitations to this work. Studies with a bigger sample size are needed to validate the role of the biomarkers in patients with axSpA and to explore the utility of combining the results of different biomarker analyses to predict treatment response. We only evaluated blood-based biomarkers for exploring ECM turnover; further research is needed to understand the ECM turnover in the affected tissues by tissue-based methods.

## Conclusion

In conclusion, this study demonstrates that ECM metabolites are associated with the pharmacodynamic effect and response to TNF-α inhibitor treatment in patients with axSpA. Therefore, selected biomarkers of ECM degradation and inflammation (C1M, C3M, C4M, and C6M), individually or together with clinical evaluation and/or MRI, shows promise as future biomarkers to assess disease activity after TNF-α inhibitor therapy.

### Supplementary Information

**Additional file 1:** **Table S1.** Patients’ characteristics of DANISH and ASIM studies in response to adalimumab based on a 50% reduction in BASDAI index.** Table S2.** Patient characteristics of DANISH and ASIM studies in response to adalimumab based on clinically important ASDAS response criteria. **Table S3.** Patients’ characteristics of DANISH and ASIM studies in response to adalimumab based on ASDAS response criteria. **Table S4.** Baseline correlations with 95% CI of the biomarkers with clinical variables in the DANISH study. **Table S5.** Baseline correlations with 95% CI of the biomarkers with clinical variables in the ASIM study. **Fig. S1.** The pharmacodynamic effect of adalimumab in the DANISH and ASIM studies in all non-significant biomarkers. ECM biomarkers percentage of change from baseline of placebo group vs. adalimumab group. A.1-F.1, DANISH study; A.2-F.2, ASIM study. Differences in % change between placebo and adalimumab group at week 12 or 6 (DANISH and ASIM, respectively). Dashed points determine the crossover to adalimumab in the placebo group. Data are presented as mean±SEM. Abbreviations: C2M, MMP(multiple)-degraded type II collagen, T2CM, MMP-1/13-mediated degradation of type II collagen, C10C, cathepsin-K mediated degradation of type X collagen CRPM, C-reactive protein metabolite, PROM, MMP-1/13-mediated degradation of prolargin, VICM, citrullinated and MMP-degraded vimentin, PRO-C2, pro-peptide of type II collagen, PRO-C3, pro-peptide of type III collagen, PRO-C6, type VI alpha-3 chain collagen.

## Data Availability

The datasets used and/or analyzed during the current study are available from the corresponding author upon reasonable request.

## Abbreviations

ADA: Adalimumab
AS: Ankylosing spondylitis
ASDAS: AS Disease Activity Score based on C-reactive protein
AxSpA: Axial spondyloarthritis
BASDAI: Bath AS Disease Activity Index
BASFI: Bath Ankylosing Spondylitis Functional Index (scale 0–10)
BASMI: Bath Ankylosing Spondylitis Metrology Index (scale 0–10)
C10C: Cathepsin-K-mediated degradation of type X collagen
C1M: Metalloproteinase (MMP)-2/913-degraded type I collagen
C2C: C-terminal of type II collagen neoepitope
C2M: MMP (multiple)-degraded type II collagen
C3M: MMP-degraded type III collagen
C4M: MMP (multiple)-degraded type IV collagen
C6M: MMP-2/9-degraded type VI collagen
CanDen: Canada-Denmark
CII: Clinical important improvement
CPa9-HNE: HNE-mediated degradation of calprotectin
CRP: C-reactive protein
CRPM: CRP metabolite
ECM: Extracellular matrix
ELISA: Enzyme-linked immunosorbent assay
HNE: Human neutrophil elastase
IDS: Immunodiagnostic systems
MI: Major improvement
MMP: Metalloproteinases
modNY criteria: Modified New York Criteria
MRI: Magnetic resonance imaging
mSASSS: Modified Stoke AS Spine Score
NI: No/low improvement
NSAIDs: Non-steroidal anti-inflammatory drugs
NTX: Type I collagen N-TELOPEPTIDES
PRO-C2: Pro-peptide of type II collagen
PRO-C3: Pro-peptide of type III collagen
PRO-C4: Type IV 7S domain collagen
PRO-C6: Type VI alpha-3 chain collagen
PROM: MMP-1- and MMP-13-mediated degradation of prolargin
PROs: Patient-reported outcomes
PsA: Psoriasis arthritis
SC: Subcutaneous
SD: Standard deviation
SIJs: Sacroiliac joints
SPARCC: Spondyloarthritis Research Consortium of Canada
SSS: Structural SIJ scores
STIR: Short tau inversion recovery
T1W: Semi-coronal T1-weighted
T2CM: MMP-1/13-mediated degradation of type II collagen
TIMP: Tissue inhibitor of MMP
TNF-α: Tumor necrosis factor α
VICM: Citrullinated and MMP-degraded vimentin
YKL-40: Human cartilage glycoprotein-39
