# Supplementary material for: Extracellular matrix turnover biomarkers reflect pharmacodynamic effects and treatment response of adalimumab in patients with axial spondyloarthritis—results from two randomized controlled trials
Source: Arthritis Res Ther. 2023 Aug 25;25:157. doi: 10.1186/s13075-023-03132-5 (PMC10463764; doi:10.1186/s13075-023-03132-5)
Supplement: Supplementary file 1 — Additional file 1: Table S1. Patients’ characteristics of DANISH and ASIM studies in response to adalimumab based on a 50% reduction in BASDAI index. Table S2. Patient characteristics of DANISH and ASIM studies in response to adalimumab based on clinically important ASDAS response criteria. Table S3. Patients’ characteristics of DANISH and ASIM studies in response to adalimumab based on ASDAS response criteria. Table S4. Baseline correlations with 95% CI of the biomarkers with clinical variables in the DANISH study. Table S5. Baseline correlations with 95% CI of the biomarkers with clinical variables in the ASIM study. Fig. S1. The pharmacodynamic effect of adalimumab in the DANISH and ASIM studies in all non-significant biomarkers. ECM biomarkers percentage of change from baseline of placebo group vs. adalimumab group. A.1-F.1, DANISH study; A.2-F.2, ASIM study. Differences in % change between placebo and adalimumab group at week 12 or 6 (DANISH and ASIM, respectively). Dashed points determine the crossover to adalimumab in the placebo group. Data are presented as mean±SEM. Abbreviations: C2M, MMP(multiple)-degraded type II collagen, T2CM, MMP-1/13-mediated degradation of type II collagen, C10C, cathepsin-K mediated degradation of type X collagen CRPM, C-reactive protein metabolite, PROM, MMP-1/13-mediated degradation of prolargin, VICM, citrullinated and MMP-degraded vimentin, PRO-C2, pro-peptide of type II collagen, PRO-C3, pro-peptide of type III collagen, PRO-C6, type VI alpha-3 chain collagen. [file 13075_2023_3132_MOESM1_ESM.docx]

**Supplementary Table S1. Patients’ characteristics of DANISH and ASIM studies in response to adalimumab based on a 50% reduction in BASDAI index***

| **Demographic feature** | DANISH | | ASIM | |
| --- | --- | --- | --- | --- |
|  | Non-responders (N=22) | Responders (N=27) | Non-responders (N=11) | Responders (N=32) |
| Age, years​ | 40.7 (11.8) | 38.6 (10.9) | 38.4 (10.3) | 36.8 (9.6) |
| Sex, male | 17 (77.3%) | 21 (77.8%) | 5 (45.5%) | 19 (59.4%) |
| HLA–B27 positive | 20 (90.9%) | 23 (85.2%) | 9 (81.8%) | 24 (75.0%) |
| Symptom duration, years | 12.3 (9.7) | 9.8 (6.9) | 13.4 (11.4) | 12.4 (11.5) |
| ASDAS, mean± SD | 3.1 (0.7) | 3.4 (0.9) | 3.4 (0.9) | 3.6 (0.8) |
| BASDAI, mean± SD (0–100 scale) | 63.3 (14.9) | 57.7 (18.2) | 6.5 (1.5) | 6.2 (1.2) |
| BASFI mean± SD | 4.7 (2.0) | 4.5 (1.9) | 5.6 (2.2) | 4.8 (2.0) |
| BASMI mean± SD | 3.1 (1.8) | 3.5 (2.2) | 3.6 (1.7) | 2.3 (2.1) |
| Fulfillment of modified New York criteria | 20 (90.9%) | 26 (96.3%) | 8 (72.7%) | 19 (59.4%) |
| SPARCC inflammation score | 5.9 (6.9) | 10.0 (12.4) | 5.0 (6.4) | 8.5 (9.3) |
| SPARCC spine inflammation score | 7.4 (9.5) | 18.7 (19.2) | 6.4 (10.2) | 8.8 (12.1) |
| CanDen spine inflammation score​ | 8.3 (11.2) | 22.2 (25.2) | 4.7 (6.9) | 7.5 (9.9) |
| mSASSS | 11.3 (13.2) | 6.7 (13.0) | 18.3 (23.3) | 4.7 (7.1) |
| C1M, ng/mL | 100.6 (114.5) | 121.8 (88.3) | 73.4 (117.2) | 82.1 (61.2) |
| C3M, ng/mL | 13.5 (3.0) | 14.6 (3.8) | 13.3 (3.0) | 13.7 (2.4) |
| C4M, ng/mL | 31.2 (9.8) | 35.3 (9.4) | 31.9 (7.9) | 33.1 (7.2) |
| C6M, ng/mL | 21.9 (9.9) | 23.1 (7.0) | 18.2 (6.5) | 22.9 (6.1) |
| CRP, mg/L | 14.9 (27.5) | 20.0 (20.1) | 9.0 (21.8) | 11.4 (11.9) |
| CRPM, ng/mL | 10.8 (3.7) | 13.3 (5.5) | 11.0 (4.0) | 14.9 (17.5) |
| PROM, ng/mL | 0.3 (0.1) | 0.2 (0.1) | 0.2 (0.1) | 0.2 (0.1) |
| VICM, ng/mL | 5.4 (4.9) | 7.0 (5.6) | 3.3 (2.6) | 3.8 (2.8) |
| CPa9-HNE, ng/mL | 78.1 (47.9) | 83.5 (33.9) | 108.9 (73.8) | 139.3 (55.1) |
| C2M, ng/mL | 21.3 (4.8) | 52.1 (169.7) | 20.1 (6.7) | 22.5 (4.8) |
| T2CM , ng/mL | 6.7 (2.2) | 6.0 (1.9) | 5.4 (0.9) | 5.4 (1.0) |
| C10C, ng/mL | 2492.7 (561.3) | 2628.5 (571.1) | 2520.6 (553.1) | 2636.1 (701.4) |
| PRO-C3, ng/mL | 11.2 (1.9) | 10.9 (2.4) | 12.0 (4.7) | 9.9 (1.6) |
| PRO-C4, ng/mL | 6912.6 (1002.3) | 6991.4 (759.1) | 6146.2 (955.0) | 6808.2 (613.4) |
| PRO-C6, ng/mL | 8.6 (3.5) | 7.8 (1.8) | 7.5 (1.5) | 7.2 (1.7) |
| PRO-C2 ng/mL | 23.1 (10.3) | 20.7 (7.4) | 18.3 (16.9) | 19.5 (13.8) |

* Except where indicated otherwise, mean ± SD is presented. Response criteria based on a 50% reduction in BASDAI index at week 12 or week 24 (DANISH and ASIM, respectively) from baseline. Abbreviations: ASDAS, Assess Disease Activity in Ankylosing Spondylitis, BASDAI, Bath Ankylosing Spondylitis Disease Activity Index (scale 0–10), BASFI, Bath Ankylosing Spondylitis Functional Index (scale 0–10), BASMI, Bath Ankylosing Spondylitis Metrology Index (scale 0–10), SPARCC, Spondyloarthritis Research Consortium of Canada, CanDen, Canada and Denmark, mSASSS, modified Stoke Ankylosing Spondylitis Score (scale 0-40).C1M, metalloproteinase (MMP)-2/913-degraded type I collagen, C3M, MMP-degraded type III collagen, C4M, MMP(multiple)-degraded type IV collagen, C6M, MMP-2/9-degraded type VI collagen, CRP, C-reactive protein, CRPM, CRP metabolite, PROM, MMP-1 and MMP-13-mediated degradation of prolargin, VICM, citrullinated and MMP-degraded vimentin, CPa9-HNE, HNE-mediated degradation of calprotectin , C2M, MMP(multiple)-degraded type II collagen, T2CM, MMP-1/13-mediated degradation of type II collagen, C10C, cathepsin-K mediated degradation of type X collagen, PRO-C3, pro-peptide of type III collagen, PRO-C4, type IV 7S domain collagen, PRO-C6, type VI alpha-3 chain collagen, PRO-C2, pro-peptide of type II collagen.

**Supplementary Table S2. Patient characteristics of DANISH and ASIM studies in response to adalimumab based on clinically important ASDAS response criteria***

| **Demographic feature** | DANISH | | | ASIM | | |
| --- | --- | --- | --- | --- | --- | --- |
|  | No clinical important improvement (N=14) | Clinically important improvement (N=14) | major improvement (N=16) | No clinical important improvement (N=12) | Clinically important improvement (N=12) | major improvement (N=19) |
| Age, years​ | 41.4 (11.0) | 39.2 (12.6) | 39.6 (10.5) | 37.3 (10.4) | 35.8 (11.1) | 37.9 (8.8) |
| Sex, male | 11 (78.6%) | 11 (78.6%) | 13 (81.2%) | 6 (50.0%) | 7 (58.3%) | 11 (57.9%) |
| HLA–B27 positive | 13 (92.9%) | 12 (85.7%) | 13 (81.2%) | 10 (83.3%) | 9 (75.0%) | 14 (73.7%) |
| Symptom duration, years | 11.5 (7.9) | 11.9 (9.0) | 9.8 (7.8) | 12.9 (11.0) | 7.5 (6.3) | 15.7 (13.1) |
| Fulfillment of modified New York criteria | 12 (85.7%) | 13 (92.9%) | 16 (100.0%) | 9 (75.0%) | 10 (83.3%) | 8 (42.1%) |
| ASDAS, mean± SD | 2.9 (0.8) | 3.0 (0.6) | 3.7 (0.8) | 3.4 (0.9) | 3.1 (0.7) | 3.9 (0.7) |
| BASDAI, mean± SD (0–100 scale) | 61.5 (17.1) | 60.7 (13.0) | 57.4 (20.5) | 6.4 (1.5) | 5.7 (1.0) | 6.5 (1.3) |
| BASFI mean± SD | 3.9 (1.8) | 4.6 (1.7) | 4.5 (2.0) | 5.5 (2.1) | 4.3 (2.1) | 5.2 (1.9) |
| BASMI mean± SD | 3.1 (1.8) | 3.4 (2.1) | 3.7 (2.2) | 3.6 (1.6) | 1.4 (1.4) | 2.9 (2.3) |
| SPARCC inflammation score | 8.6 (7.8) | 4.1 (8.6) | 11.8 (13.2) | 4.6 (6.3) | 12.8 (11.8) | 6.1 (6.2) |
| SPARCC spine inflammation score | 9.9 (10.6) | 10.9 (12.1) | 21.6 (23.0) | 7.4 (10.4) | 6.8 (9.7) | 9.5 (13.6) |
| CanDen spine inflammation score​ | 7.5 (8.5) | 10.1 (10.3) | 28.9 (30.3) | 5.4 (7.0) | 5.5 (7.3) | 8.4 (11.4) |
| mSASSS | 7.4 (13.5) | 9.4 (10.2) | 8.1 (15.7) | 17.8 (22.3) | 3.2 (5.7) | 5.2 (7.8) |
| C1M, ng/mL | 55.8 (33.1) | 96.2 (102.3) | 162.4 (92.6) | 69.3 (112.6) | 62.6 (50.7) | 97.5 (64.6) |
| C3M, ng/mL | 12.2 (2.1) | 13.9 (3.2) | 16.0 (4.0) | 13.3 (2.9) | 13.1 (2.8) | 14.0 (2.2) |
| C4M, ng/mL | 26.8 (5.1) | 30.7 (6.2) | 39.9 (9.3) | 31.5 (7.7) | 31.2 (8.1) | 34.6 (6.5) |
| C6M, ng/mL | 17.6 (6.3) | 21.3 (8.3) | 26.7 (6.6) | 18.5 (6.3) | 23.2 (7.3) | 22.7 (5.6) |
| CRP, mg/L | 4.8 (5.4) | 11.3 (21.1) | 30.7 (19.9) | 8.5 (20.8) | 8.0 (10.5) | 13.9 (12.6) |
| CRPM, ng/mL | 11.0 (3.7) | 10.8 (2.7) | 14.7 (6.6) | 11.7 (4.7) | 12.1 (5.1) | 16.5 (22.3) |
| PROM, ng/mL | 0.2 (0.1) | 0.3 (0.1) | 0.2 (0.1) | 0.2 (0.1) | 0.2 (0.1) | 0.3 (0.1) |
| VICM, ng/mL | 4.5 (3.0) | 4.9 (4.2) | 8.8 (6.3) | 3.2 (2.5) | 4.3 (3.9) | 3.5 (2.1) |
| CPa9-HNE, ng/mL | 72.5 (29.4) | 60.5 (23.6) | 96.9 (35.9) | 111.8 (71.0) | 133.2 (42.1) | 142.9 (64.1) |
| C2M, ng/mL | 22.1 (5.3) | 19.4 (2.8) | 74.5 (220.5) | 20.2 (6.4) | 23.0 (4.5) | 22.3 (5.1) |
| T2CM , ng/mL | 6.2 (1.4) | 6.9 (2.6) | 5.9 (2.1) | 5.4 (0.9) | 5.4 (1.2) | 5.4 (1.0) |
| C10C, ng/mL | 2607.9 (578.8) | 2524.1 (468.0) | 2666.2 (627.7) | 2432.3 (609.7) | 2787.7 (818.4) | 2602.2 (587.1) |
| PRO-C3, ng/mL | 10.3 (1.2) | 11.6 (2.5) | 10.7 (2.1) | 11.8 (4.5) | 10.2 (1.7) | 9.8 (1.6) |
| PRO-C4, ng/mL | 6522.2 (568.8) | 7089.3 (1133.3) | 7167.5 (673.6) | 6118.3 (915.7) | 6716.8 (638.5) | 6918.4 (570.3) |
| PRO-C6, ng/mL | 8.2 (3.0) | 8.1 (3.4) | 8.2 (2.0) | 7.3 (1.8) | 7.3 (1.4) | 7.3 (1.8) |
| PRO-C2 ng/mL | 21.8 (9.5) | 24.2 (10.3) | 21.2 (7.9) | 18.3 (16.1) | 18.8 (15.7) | 19.9 (13.3) |

* Except where indicated otherwise, mean ± SD is presented. Response criteria based on ASDAS Δ at week 12 or week 24 (DANISH and ASIM, respectively) from baseline: No clinical important improvement, ASDAS Δ <1.1, clinically important improvement, 1.1≤ΔASDAS<2.0, major improvement, Δ ≥2. Abbreviations: ASDAS, Assess Disease Activity in Ankylosing Spondylitis, BASDAI, Bath Ankylosing Spondylitis Disease Activity Index (scale 0–10), BASFI, Bath Ankylosing Spondylitis Functional Index (scale 0–10), BASMI, Bath Ankylosing Spondylitis Metrology Index (scale 0–10), SPARCC, Spondyloarthritis Research Consortium of Canada, CanDen, Canada and Denmark, mSASSS, modified Stoke Ankylosing Spondylitis Score (scale 0-40).C1M, metalloproteinase (MMP)-2/913-degraded type I collagen, C3M, MMP-degraded type III collagen, C4M, MMP(multiple)-degraded type IV collagen, C6M, MMP-2/9-degraded type VI collagen, CRP, C-reactive protein, CRPM, CRP metabolite, PROM, MMP-1 and MMP-13-mediated degradation of prolargin, VICM, citrullinated and MMP-degraded vimentin, CPa9-HNE, HNE-mediated degradation of calprotectin , C2M, MMP(multiple)-degraded type II collagen, T2CM, MMP-1/13-mediated degradation of type II collagen, C10C, cathepsin-K mediated degradation of type X collagen, PRO-C3, pro-peptide of type III collagen, PRO-C4, type IV 7S domain collagen, PRO-C6, type VI alpha-3 chain collagen, PRO-C2, pro-peptide of type II collagen.

**Supplementary Table S3. Patients’ characteristics of DANISH and ASIM studies in response to adalimumab based on ASDAS response criteria.**

| **Demographic feature** | DANISH | | ASIM | |
| --- | --- | --- | --- | --- |
|  | **No clinical important improvement (N=28)** | **≥ important clinical improvement (N=16)** | **No clinical important improvement (N=24)** | **≥ important clinical improvement (N=19)** |
| Age, years​ | 40.3 (11.7) | 39.6 (10.5) | 36.6 (10.5) | 37.9 (8.8) |
| Sex, male | 22 (78.6%) | 13 (81.2%) | 13 (54.2%) | 11 (57.9%) |
| HLA–B27 positive | 25 (89.3%) | 13 (81.2%) | 19 (79.2%) | 14 (73.7%) |
| Symptom duration, years | 11.7 (8.3) | 9.8 (7.8) | 10.2 (9.2) | 15.7 (13.1) |
| Fulfillment of modified New York criteria | 25 (89.3%) | 16 (100.0%) | 19 (79.2%) | 8 (42.1%) |
| ASDAS, mean± SD | 3.0 (0.7) | 3.7 (0.8) | 3.2 (0.8) | 3.9 (0.7) |
| BASDAI, mean± SD (0–100 scale) | 61.1 (14.9) | 57.4 (20.5) | 6.1 (1.3) | 6.5 (1.3) |
| BASFI mean± SD | 4.3 (1.8) | 4.5 (2.0) | 4.9 (2.2) | 5.2 (1.9) |
| BASMI mean± SD | 3.2 (1.9) | 3.7 (2.2) | 2.5 (1.9) | 2.9 (2.3) |
| SPARCC inflammation score | 6.3 (8.4) | 11.8 (13.2) | 8.7 (10.1) | 6.1 (6.2) |
| SPARCC spine inflammation score | 10.4 (11.2) | 21.6 (23.0) | 7.1 (9.8) | 9.5 (13.6) |
| CanDen spine inflammation score​ | 8.8 (9.4) | 28.9 (30.3) | 5.5 (7.0) | 8.4 (11.4) |
| mSASSS | 8.5 (11.7) | 8.1 (15.7) | 10.5 (17.6) | 5.2 (7.8) |
| C1M, ng/mL | 76.0 (77.4) | 162.4 (92.6) | 66.0 (85.5) | 97.5 (64.6) |
| C3M ng/mL | 13.0 (2.8) | 16.0 (4.0) | 13.2 (2.8) | 14.0 (2.2) |
| C4M, ng/mL | 28.8 (5.9) | 39.9 (9.3) | 31.4 (7.7) | 34.6 (6.5) |
| C6M, ng/mL | 19.4 (7.5) | 26.7 (6.6) | 20.9 (7.1) | 22.7 (5.6) |
| CRP, mg/lL | 8.1 (15.4) | 30.7 (19.9) | 8.2 (16.1) | 13.9 (12.6) |
| CRPM, ng/mL | 10.9 (3.1) | 14.7 (6.6) | 11.9 (4.8) | 16.5 (22.3) |
| PROM, ng/mL | 0.3 (0.1) | 0.2 (0.1) | 0.2 (0.1) | 0.3 (0.1) |
| VICM, ng/mL | 4.7 (3.6) | 8.8 (6.3) | 3.7 (3.2) | 3.5 (2.1) |
| CPa9-HNE, ng/mL | 66.5 (26.9) | 96.9 (35.9) | 122.5 (58.1) | 142.9 (64.1) |
| C2M, ng/mL | 20.8 (4.4) | 74.5 (220.5) | 21.6 (5.6) | 22.3 (5.1) |
| T2CM, ng/mL | 6.5 (2.1) | 5.9 (2.1) | 5.4 (1.0) | 5.4 (1.0) |
| C10C, ng/mL | 2566.0 (518.2) | 2666.2 (627.7) | 2610.0 (728.8) | 2602.2 (587.1) |
| PRO-C3, ng/mL | 11.0 (2.0) | 10.7 (2.1) | 11.0 (3.5) | 9.8 (1.6) |
| PRO-C4, ng/mL | 6805.7 (926.1) | 7167.5 (673.6) | 6417.6 (830.3) | 6918.4 (570.3) |
| PRO-C6, ng/mL | 8.2 (3.2) | 8.2 (2.0) | 7.3 (1.6) | 7.3 (1.8) |
| PRO-C2 ng/mL | 23.0 (9.8) | 21.2 (7.9) | 18.5 (15.5) | 19.9 (13.3) |

* Except where indicated otherwise, mean ± SD is presented. Response criteria based on ASDAS Δ at week 12 or 6 (DANISH and ASIM, respectively) from baseline: no clinical important improvement (NI), Δ <1.1, at least clinical important improvement (≥CII), Δ ≥1.1. Abbreviations: ASDAS, Assess Disease Activity in Ankylosing Spondylitis, BASDAI, Bath Ankylosing Spondylitis Disease Activity Index (scale 0–10), BASFI, Bath Ankylosing Spondylitis Functional Index (scale 0–10), BASMI, Bath Ankylosing Spondylitis Metrology Index (scale 0–10), SPARCC, Spondyloarthritis Research Consortium of Canada, CanDen, Canada and Denmark, mSASSS, modified Stoke Ankylosing Spondylitis Score (scale 0-40).C1M, metalloproteinase (MMP)-2/913-degraded type I collagen, C3M, MMP-degraded type III collagen, C4M, MMP(multiple)-degraded type IV collagen, C6M, MMP-2/9-degraded type VI collagen, CRP, C-reactive protein, CRPM, CRP metabolite, PROM, MMP-1 and MMP-13-mediated degradation of prolargin, VICM, citrullinated and MMP-degraded vimentin, CPa9-HNE, HNE-mediated degradation of calprotectin , C2M, MMP(multiple)-degraded type II collagen, T2CM, MMP-1/13-mediated degradation of type II collagen, C10C, cathepsin-K mediated degradation of type X collagen, PRO-C3, pro-peptide of type III collagen, PRO-C4, type IV 7S domain collagen, PRO-C6, type VI alpha-3 chain collagen, PRO-C2, pro-peptide of type II collagen.

**Supplementary Table S4. Baseline correlations with 95% CI of the biomarkers with clinical variables in the DANISH study.**

| DANISH | **Age** | **Symptom**  **duration** | **ASDAS** | **BASDAI** | **BASFI** | **BASMI** | **SPARCC**  **SSS**  **inflammation**  **score** | **SPARCC**  **SSS**  **fat**  **score** | **SPARCC**  **SSS**  **erosion score** | **SPARCC**  **SSS**  **backfill score** | **SPARCC**  **SSS**  **ankylosis score** | **SPARCC spine inflammation score** | **CanDen**  **Spine**  **inflammation score** | **CanDen**  **spine**  **fat score** | **mSASSS** |
| --- | --- | --- | --- | --- | --- | --- | --- | --- | --- | --- | --- | --- | --- | --- | --- |
| **C1M** | -0.03 [-0.34, 0.28] | 0.04 [-0.27, 0.34] | ***0.68 [0.47, 0.82]*** | -0.02 [-0.33, 0.29] | 0.26 [-0.05, 0.53] | 0.20 [-0.12, 0.48] | -0.07 [-0.37, 0.24] | 0.32 [0.01, 0.57] | -0.26 [-0.52, 0.06] | -0.05 [-0.35, 0.26] | 0.27 [-0.04, 0.53] | 0.36 [0.06, 0.60] | 0.49 [0.21, 0.69] | 0.23 [-0.08, 0.50] | 0.17 [-0.15, 0.45] |
| **C3M** | -0.16 [-0.45, 0.15] | -0.09 [-0.39, 0.22] | ***0.44 [0.15, 0.66]*** | 0.05 [-0.26, 0.35] | 0.32 [0.01, 0.57] | 0.07 [-0.25, 0.37] | -0.01 [-0.31, 0.30] | 0.01 [-0.30, 0.31] | -0.18 [-0.46, 0.14] | -0.01 [-0.31, 0.30] | 0.19 [-0.12, 0.47] | 0.04 [-0.27, 0.35] | 0.16 [-0.16, 0.45] | 0.18 [-0.14, 0.46] | -0.01 [-0.32, 0.30] |
| **C4M** | -0.06 [-0.36, 0.26] | -0.09 [-0.39, 0.22] | ***0.53 [0.27, 0.72]*** | 0.01 [-0.29, 0.32] | 0.29 [-0.02, 0.55] | 0.14 [-0.17, 0.43] | -0.04 [-0.35, 0.27] | 0.21 [-0.10, 0.49] | -0.18 [-0.47, 0.13] | -0.09 [-0.39, 0.22] | 0.22 [-0.10, 0.49] | 0.32 [0.01, 0.57] | 0.45 [0.17, 0.67] | 0.20 [-0.11, 0.48] | 0.04 [-0.27, 0.35] |
| **C6M** | -0.07 [-0.37, 0.24] | -0.02 [-0.33, 0.29] | ***0.63 [0.40, 0.79]*** | 0.07 [-0.24, 0.37] | 0.25 [-0.06, 0.52] | 0.24 [-0.07, 0.51] | -0.11 [-0.40, 0.20] | 0.21 [-0.10, 0.49] | -0.31 [-0.57, -0.01] | -0.00 [-0.31, 0.31] | 0.32 [0.01, 0.57] | 0.18 [-0.13, 0.46] | 0.36 [0.06, 0.60] | 0.23 [-0.08, 0.50] | 0.19 [-0.12, 0.47] |
| **CRP** | 0.00 [-0.31, 0.31] | 0.03 [-0.28, 0.33] | ***0.72 [0.53, 0.84]*** | -0.02 [-0.33, 0.29] | 0.22 [-0.09, 0.50] | 0.14 [-0.17, 0.43] | -0.11 [-0.41, 0.20] | 0.33 [0.02, 0.58] | -0.34 [-0.59, -0.04] | -0.00 [-0.31, 0.30] | 0.37 [0.07, 0.61] | 0.39 [0.09, 0.62] | 0.52 [0.25, 0.71] | 0.29 [-0.02, 0.55] | 0.13 [-0.18, 0.42] |
| **CRPM** | -0.06 [-0.36, 0.25] | -0.03 [-0.33, 0.28] | 0.16 [-0.15, 0.45] | -0.02 [-0.32, 0.29] | -0.01 [-0.31, 0.30] | 0.05 [-0.26, 0.35] | -0.08 [-0.38, 0.24] | 0.03 [-0.28, 0.33] | -0.09 [-0.39, 0.23] | 0.03 [-0.28, 0.33] | 0.13 [-0.18, 0.42] | -0.04 [-0.35, 0.27] | 0.05 [-0.26, 0.36] | 0.13 [-0.19, 0.42] | -0.19 [-0.47, 0.13] |
| **PROM** | 0.10 [-0.21, 0.40] | -0.02 [-0.33, 0.29] | 0.15 [-0.16, 0.44] | 0.17 [-0.14, 0.46] | 0.24 [-0.07, 0.51] | 0.26 [-0.05, 0.53] | -0.23 [-0.50, 0.08] | -0.02 [-0.33, 0.29] | -0.32 [-0.57, -0.01] | -0.30 [-0.56, 0.01] | 0.24 [-0.08, 0.51] | -0.06 [-0.36, 0.25] | -0.05 [-0.35, 0.27] | 0.06 [-0.26, 0.36] | ***0.33 [0.02, 0.58]*** |
| **VICM** | -0.06 [-0.36, 0.25] | -0.18 [-0.46, 0.14] | ***0.22 [-0.09, 0.50]*** | -0.23 [-0.50, 0.08] | -0.01 [-0.32, 0.30] | -0.17 [-0.45, 0.15] | -0.02 [-0.32, 0.29] | 0.26 [-0.05, 0.53] | 0.12 [-0.20, 0.41] | -0.15 [-0.43, 0.17] | -0.04 [-0.35, 0.27] | 0.16 [-0.16, 0.44] | 0.15 [-0.16, 0.44] | -0.07 [-0.37, 0.25] | -0.14 [-0.43, 0.17] |
| **CPa9-HNE** | -0.07 [-0.37, 0.25] | -0.03 [-0.33, 0.28] | ***0.64 [0.42, 0.79]*** | 0.07 [-0.24, 0.37] | 0.21 [-0.11, 0.49] | 0.03 [-0.28, 0.33] | 0.11 [-0.21, 0.40] | 0.26 [-0.05, 0.53] | -0.07 [-0.37, 0.24] | 0.08 [-0.23, 0.38] | 0.18 [-0.14, 0.46] | 0.37 [0.07, 0.61] | 0.50 [0.22, 0.70] | 0.27 [-0.04, 0.54] | 0.05 [-0.27, 0.35] |
| **C2M** | 0.32 [0.02, 0.57] | 0.41 [0.11, 0.63] | -0.04 [-0.35, 0.27] | -0.15 [-0.44, 0.16] | -0.12 [-0.41, 0.20] | -0.07 [-0.37, 0.24] | -0.06 [-0.36, 0.25] | -0.10 [-0.39, 0.22] | 0.01 [-0.30, 0.31] | 0.06 [-0.25, 0.36] | 0.29 [-0.02, 0.55] | 0.15 [-0.16, 0.44] | 0.17 [-0.15, 0.45] | 0.23 [-0.08, 0.51] | 0.33 [0.02, 0.58] |
| **T2CM** | 0.11 [-0.20, 0.40] | 0.03 [-0.28, 0.33] | -0.09 [-0.39, 0.22] | 0.19 [-0.12, 0.47] | 0.18 [-0.13, 0.46] | 0.05 [-0.26, 0.35] | -0.03 [-0.33, 0.28] | -0.21 [-0.48, 0.11] | 0.01 [-0.30, 0.32] | 0.13 [-0.18, 0.42] | -0.08 [-0.38, 0.23] | -0.27 [-0.53, 0.04] | -0.25 [-0.52, 0.06] | -0.08 [-0.38, 0.23] | 0.16 [-0.15, 0.45] |
| **C10C** | 0.36 [0.06, 0.60] | 0.11 [-0.21, 0.40] | -0.18 [-0.46, 0.13] | -0.12 [-0.41, 0.20] | 0.12 [-0.19, 0.41] | -0.00 [-0.31, 0.30] | 0.10 [-0.22, 0.39] | -0.22 [-0.50, 0.09] | 0.08 [-0.23, 0.38] | 0.03 [-0.28, 0.33] | -0.24 [-0.51, 0.07] | 0.09 [-0.23, 0.38] | -0.04 [-0.35, 0.27] | -0.07 [-0.37, 0.24] | -0.15 [-0.44, 0.17] |
| **PRO-C3** | 0.11 [-0.20, 0.41] | -0.01 [-0.32, 0.29] | -0.01 [-0.32, 0.29] | 0.04 [-0.27, 0.34] | 0.04 [-0.27, 0.34] | -0.15 [-0.44, 0.17] | -0.23 [-0.50, 0.08] | 0.05 [-0.26, 0.36] | -0.10 [-0.39, 0.22] | -0.30 [-0.55, 0.01] | -0.01 [-0.32, 0.30] | -0.01 [-0.32, 0.30] | -0.05 [-0.35, 0.26] | -0.06 [-0.36, 0.25] | 0.10 [-0.22, 0.39] |
| **PRO-C4** | 0.07 [-0.24, 0.37] | -0.07 [-0.37, 0.25] | 0.23 [-0.09, 0.50] | -0.12 [-0.41, 0.20] | 0.19 [-0.12, 0.47] | 0.04 [-0.27, 0.35] | -0.14 [-0.43, 0.18] | 0.10 [-0.22, 0.39] | -0.20 [-0.48, 0.11] | -0.14 [-0.43, 0.18] | -0.01 [-0.31, 0.30] | 0.14 [-0.17, 0.43] | 0.13 [-0.18, 0.42] | 0.11 [-0.21, 0.40] | -0.03 [-0.34, 0.28] |
| **PRO-C6** | -0.03 [-0.33, 0.28] | -0.18 [-0.47, 0.13] | 0.11 [-0.20, 0.41] | 0.28 [-0.04, 0.54] | 0.22 [-0.10, 0.49] | 0.29 [-0.02, 0.55] | -0.03 [-0.33, 0.28] | 0.19 [-0.12, 0.47] | -0.01 [-0.31, 0.30] | -0.09 [-0.39, 0.22] | 0.05 [-0.26, 0.35] | -0.14 [-0.43, 0.18] | -0.07 [-0.37, 0.24] | -0.08 [-0.38, 0.23] | 0.24 [-0.07, 0.51] |
| **PRO-C2** | -0.23 [-0.50, 0.08] | -0.08 [-0.38, 0.23] | 0.01 [-0.29, 0.32] | -0.05 [-0.35, 0.26] | 0.21 [-0.10, 0.49] | -0.01 [-0.32, 0.30] | 0.04 [-0.27, 0.34] | -0.15 [-0.44, 0.16] | -0.06 [-0.37, 0.25] | -0.40 [-0.63, -0.11] | 0.18 [-0.13, 0.47] | 0.07 [-0.24, 0.37] | 0.00 [-0.30, 0.31] | -0.04 [-0.34, 0.27] | -0.10 [-0.39, 0.22] |
|  |  |  |  |  |  |  | Correlation coefficient | | | |  |  |  |  |  |
|  |  |  |  |  | 1 | 0.5 | 0.3 | 0 | -0.3 | -0.5 | -1 |  |  |  |  |

Spearman ́s correlation between serological metabolites and clinical scores; Spearman ́s rho (ρ) is shown. The bold and italic ρ determines the correlations that reached significance with a p-value<0.01. Abbreviations: ASDAS, Assess Disease Activity in Ankylosing Spondylitis, BASDAI, Bath Ankylosing Spondylitis Disease Activity Index (scale 0–10), BASFI, Bath Ankylosing Spondylitis Functional Index (scale 0–10), BASMI, Bath Ankylosing Spondylitis Metrology Index (scale 0–10), SPARC, Spondyloarthritis Research Consortium of Canada, SSS, SI joint structural lesion score, CanDen, Canada and Denmark, mSASS, modified Stoke Ankylosing Spondylitis Score(scale 0-40), C1M, metalloproteinase (MMP)-2/913-degraded type I collagen, C3M, MMP-degraded type III collagen, C4M, MMP(multiple)-degraded type IV collagen, C6M, MMP-2/9-degraded type VI collagen, CRP, C-reactive protein, CRPM, CRP metabolite, PROM, MMP-1 and MMP-13-mediated degradation of prolargin, VICM, citrullinated and MMP-degraded vimentin, CPa9-HNE, HNE-mediated degradation of calprotectin , C2M, MMP(multiple)-degraded type II collagen, T2CM, MMP-1/13-mediated degradation of type II collagen, C10C, cathepsin-K mediated degradation of type X collagen PRO-C3, pro-peptide of type III collagen ,PRO-C4, type IV 7S domain collagen, PRO-C6, type VI alpha-3 chain collagen, PRO-C2, pro-peptide of type II collagen.

| ASIM | **Age** | **Symptom**  **duration** | **ASDAS** | **BASDAI** | **BASFI** | | **BASMI** | **SPARCC**  **SSS**  **inflammation**  **score** | **SPARCC**  **SSS**  **fat**  **score** | **SPARCC**  **SSS**  **erosion**  **score** | **SPARCC**  **SSS**  **backfill score** | **SPARCC**  **SSS**  **ankylosis score** | **SPARCC spine inflammation**  **score** | **CanDen**  **Spine**  **inflammation score** | **CanDen**  **spine**  **fat**  **score** | **mSASSS** |
| --- | --- | --- | --- | --- | --- | --- | --- | --- | --- | --- | --- | --- | --- | --- | --- | --- |
| **C1M** | 0.05 [-0.25, 0.34] | 0.07 [-0.23, 0.36] | ***0.70 [0.51, 0.83]*** | 0.15 [-0.16, 0.43] | | 0.21 [-0.09, 0.48] | 0.09 [-0.22, 0.37] | 0.11 [-0.19, 0.39] | -0.07 [-0.36, 0.23] | 0.13 [-0.18, 0.41] | -0.14 [-0.42, 0.16] | -0.02 [-0.32, 0.28] | 0.10 [-0.20, 0.39] | 0.16 [-0.14, 0.44] | 0.11 [-0.20, 0.39] | -0.00 [-0.30, 0.29] |
| **C3M** | -0.03 [-0.32, 0.27] | -0.02 [-0.32, 0.28] | ***0.36 [0.07, 0.59]*** | -0.11 [-0.39, 0.20] | | -0.04 [-0.33, 0.26] | -0.11 [-0.39, 0.19] | 0.09 [-0.21, 0.38] | -0.00 [-0.30, 0.29] | 0.32 [0.02, 0.56] | -0.07 [-0.36, 0.23] | 0.03 [-0.27, 0.32] | 0.07 [-0.23, 0.36] | 0.12 [-0.18, 0.41] | 0.16 [-0.14, 0.44] | -0.03 [-0.33, 0.27] |
| **C4M** | 0.03 [-0.27, 0.32] | 0.11 [-0.20, 0.39] | ***0.51 [0.25, 0.70]*** | 0.00 [-0.29, 0.30] | | 0.16 [-0.15, 0.43] | 0.07 [-0.23, 0.36] | 0.10 [-0.20, 0.39] | 0.10 [-0.21, 0.38] | 0.14 [-0.16, 0.42] | -0.12 [-0.40, 0.19] | 0.20 [-0.10, 0.47] | 0.15 [-0.15, 0.43] | 0.21 [-0.09, 0.48] | 0.26 [-0.04, 0.51] | 0.13 [-0.18, 0.41] |
| **C6M** | -0.21 [-0.47, 0.10] | -0.01 [-0.31, 0.28] | ***0.48 [0.21, 0.68]*** | -0.06 [-0.35, 0.24] | | 0.10 [-0.20, 0.38] | 0.09 [-0.22, 0.37] | 0.12 [-0.18, 0.41] | 0.11 [-0.19, 0.40] | 0.05 [-0.25, 0.34] | -0.19 [-0.46, 0.11] | 0.17 [-0.13, 0.45] | 0.13 [-0.18, 0.41] | 0.16 [-0.14, 0.44] | 0.13 [-0.17, 0.41] | 0.05 [-0.25, 0.34] |
| **CRP** | -0.11 [-0.39, 0.19] | 0.06 [-0.24, 0.35] | ***0.70 [0.51, 0.83]*** | 0.12 [-0.19, 0.40] | | 0.22 [-0.08, 0.49] | 0.12 [-0.19, 0.40] | 0.02 [-0.28, 0.32] | -0.08 [-0.37, 0.22] | 0.01 [-0.29, 0.31] | -0.12 [-0.40, 0.19] | 0.13 [-0.17, 0.41] | 0.12 [-0.19, 0.40] | 0.17 [-0.13, 0.45] | 0.04 [-0.26, 0.33] | 0.04 [-0.26, 0.33] |
| **CRPM** | -0.09 [-0.38, 0.21] | -0.22 [-0.49, 0.08] | 0.27 [-0.03, 0.53] | 0.10 [-0.20, 0.39] | | 0.04 [-0.26, 0.33] | -0.19 [-0.46, 0.11] | 0.28 [-0.02, 0.53] | -0.17 [-0.45, 0.13] | ***0.44 [0.17, 0.65]*** | 0.06 [-0.24, 0.35] | -0.23 [-0.49, 0.07] | -0.06 [-0.35, 0.24] | -0.03 [-0.33, 0.27] | -0.05 [-0.34, 0.25] | -0.26 [-0.51, 0.04] |
| **PROM** | 0.26 [-0.04, 0.52] | 0.29 [-0.00, 0.54] | 0.13 [-0.18, 0.41] | -0.06 [-0.35, 0.24] | | 0.06 [-0.25, 0.35] | -0.04 [-0.34, 0.26] | -0.03 [-0.33, 0.27] | 0.05 [-0.26, 0.34] | -0.09 [-0.37, 0.21] | -0.06 [-0.35, 0.25] | 0.09 [-0.21, 0.38] | 0.21 [-0.09, 0.48] | 0.25 [-0.05, 0.51] | 0.18 [-0.12, 0.45] | 0.05 [-0.25, 0.34] |
| **VICM** | -0.12 [-0.40, 0.18] | -0.03 [-0.32, 0.27] | 0.14 [-0.16, 0.42] | 0.06 [-0.25, 0.35] | | 0.24 [-0.06, 0.50] | 0.21 [-0.09, 0.48] | 0.15 [-0.15, 0.43] | 0.32 [0.02, 0.56] | 0.04 [-0.26, 0.33] | -0.11 [-0.39, 0.19] | 0.20 [-0.10, 0.47] | -0.15 [-0.43, 0.15] | -0.12 [-0.40, 0.19] | 0.16 [-0.15, 0.43] | -0.01 [-0.31, 0.28] |
| **CPa9-HNE** | ***-0.34 [-0.58, -0.05]*** | -0.15 [-0.43, 0.15] | ***0.51 [0.25, 0.70]*** | 0.04 [-0.26, 0.33] | | 0.07 [-0.24, 0.36] | 0.05 [-0.25, 0.35] | 0.04 [-0.26, 0.34] | -0.06 [-0.35, 0.24] | -0.03 [-0.32, 0.27] | -0.15 [-0.43, 0.15] | 0.07 [-0.23, 0.36] | -0.09 [-0.38, 0.21] | -0.05 [-0.34, 0.25] | -0.05 [-0.34, 0.25] | -0.03 [-0.32, 0.27] |
| **C2M** | -0.12 [-0.40, 0.19] | 0.11 [-0.19, 0.40] | -0.13 [-0.41, 0.17] | -0.23 [-0.49, 0.07] | | -0.03 [-0.33, 0.27] | -0.13 [-0.41, 0.18] | 0.22 [-0.08, 0.49] | 0.25 [-0.06, 0.51] | 0.18 [-0.12, 0.45] | 0.08 [-0.22, 0.37] | 0.12 [-0.18, 0.40] | -0.11 [-0.39, 0.19] | -0.09 [-0.38, 0.21] | 0.12 [-0.19, 0.40] | -0.04 [-0.34, 0.26] |
| **T2CM** | 0.23 [-0.07, 0.50] | 0.17 [-0.13, 0.45] | 0.11 [-0.20, 0.39] | 0.20 [-0.11, 0.47] | | -0.00 [-0.30, 0.29] | -0.05 [-0.34, 0.25] | -0.09 [-0.38, 0.21] | -0.07 [-0.36, 0.23] | 0.02 [-0.28, 0.32] | 0.13 [-0.17, 0.41] | -0.09 [-0.38, 0.21] | -0.03 [-0.33, 0.27] | -0.05 [-0.35, 0.25] | 0.02 [-0.28, 0.31] | -0.10 [-0.39, 0.20] |
| **C10C** | 0.10 [-0.20, 0.39] | -0.03 [-0.32, 0.27] | 0.14 [-0.16, 0.42] | 0.11 [-0.19, 0.40] | | 0.06 [-0.24, 0.35] | -0.17 [-0.44, 0.13] | 0.04 [-0.26, 0.33] | ***-0.31 [-0.55, -0.01]*** | -0.03 [-0.32, 0.27] | -0.10 [-0.38, 0.21] | -0.21 [-0.48, 0.09] | -0.10 [-0.39, 0.20] | -0.11 [-0.39, 0.20] | -0.22 [-0.48, 0.08] | 0.08 [-0.22, 0.37] |
| **PRO-C3** | -0.26 [-0.51, 0.04] | -0.21 [-0.48, 0.09] | 0.06 [-0.24, 0.35] | -0.00 [-0.30, 0.29] | | -0.07 [-0.36, 0.23] | -0.09 [-0.38, 0.21] | 0.07 [-0.23, 0.36] | -0.09 [-0.37, 0.22] | 0.25 [-0.06, 0.51] | 0.01 [-0.29, 0.30] | -0.23 [-0.49, 0.07] | -0.04 [-0.33, 0.26] | -0.02 [-0.32, 0.28] | -0.09 [-0.38, 0.21] | ***-0.31 [-0.56, -0.02]*** |
| **PRO-C4** | -0.13 [-0.41, 0.17] | 0.10 [-0.21, 0.38] | 0.36 [0.07, 0.59] | 0.03 [-0.27, 0.32] | | 0.24 [-0.06, 0.50] | -0.09 [-0.38, 0.21] | 0.12 [-0.18, 0.40] | -0.02 [-0.31, 0.28] | 0.28 [-0.02, 0.53] | -0.08 [-0.37, 0.22] | -0.16 [-0.44, 0.14] | -0.09 [-0.38, 0.21] | -0.07 [-0.36, 0.23] | 0.14 [-0.17, 0.42] | -0.19 [-0.46, 0.12] |
| **PRO-C6** | 0.20 [-0.10, 0.47] | -0.18 [-0.45, 0.12] | 0.05 [-0.25, 0.34] | 0.04 [-0.26, 0.34] | | -0.00 [-0.30, 0.29] | -0.16 [-0.44, 0.14] | ***0.31 [0.01, 0.55]*** | -0.06 [-0.35, 0.24] | ***0.36 [0.07, 0.60]*** | 0.02 [-0.28, 0.32] | ***-0.43 [-0.65, -0.16]*** | -0.21 [-0.48, 0.09] | -0.21 [-0.47, 0.10] | -0.04 [-0.34, 0.26] | -0.29 [-0.54, 0.01] |
| **PRO-C2** | -0.05 [-0.35, 0.25] | 0.08 [-0.22, 0.37] | -0.09 [-0.37, 0.21] | -0.19 [-0.46, 0.11] | | -0.16 [-0.44, 0.14] | -0.09 [-0.38, 0.21] | 0.00 [-0.30, 0.30] | -0.06 [-0.35, 0.24] | 0.09 [-0.21, 0.37] | 0.10 [-0.20, 0.39] | 0.17 [-0.13, 0.45] | 0.07 [-0.23, 0.36] | 0.12 [-0.18, 0.40] | 0.10 [-0.20, 0.38] | -0.03 [-0.33, 0.26] |
|  |  |  |  |  | |  |  | Correlation coefficient | | | |  |  |  |  |  |
|  |  |  |  |  | | 1 | 0.5 | 0.3 | 0 | -0.3 | -0.5 | -1 |  |  |  |  |

**Supplementary Table S5. Baseline correlations with 95% CI of the biomarkers with clinical variables in the ASIM study.**

Spearman ́s correlation between serological metabolites and clinical; Spearman ́s rho (ρ) is shown. The bold and italic ρ determines the correlations that reached significance with a p-value<0.01. Abbreviations: ASDAS, Assess Disease Activity in Ankylosing Spondylitis, BASDAI, Bath Ankylosing Spondylitis Disease Activity Index (scale 0–10), BASFI, Bath Ankylosing Spondylitis Functional Index (scale 0–10), BASMI, Bath Ankylosing Spondylitis Metrology Index (scale 0–10), SPARC, Spondyloarthritis Research Consortium of Canada, SSS, SI joint structural lesion score, CanDen, Canada and Denmark, mSASS, modified Stoke Ankylosing Spondylitis Score(scale 0-40), C1M, metalloproteinase (MMP)-2/913-degraded type I collagen, C3M, MMP-degraded type III collagen, C4M, MMP(multiple)-degraded type IV collagen, C6M, MMP-2/9-degraded type VI collagen, CRP, C-reactive protein, CRPM, CRP metabolite, PROM, MMP-1 and MMP-13-mediated degradation of prolargin, VICM, citrullinated and MMP-degraded vimentin, CPa9-HNE, HNE-mediated degradation of calprotectin , C2M, MMP(multiple)-degraded type II collagen, T2CM, MMP-1/13-mediated degradation of type II collagen, C10C, cathepsin-K mediated degradation of type X collagen PRO-C3, pro-peptide of type III collagen ,PRO-C4, type IV 7S domain collagen, PRO-C6, type VI alpha-3 chain collagen, PRO-C2, pro-peptide of type II collagen.

**Supplementary Figure S1. The pharmacodynamic effect of adalimumab in the DANISH and ASIM studies in all non-significant biomarkers**. ECM biomarkers percentage of change from baseline of placebo group vs. adalimumab group. A.1-F.1, DANISH study; A.2-F.2, ASIM study. Differences in % change between placebo and adalimumab group at week 12 or 6 (DANISH and ASIM, respectively). Dashed points determine the crossover to adalimumab in the placebo group. Data are presented as mean ±SEM. Abbreviations: C2M, MMP(multiple)-degraded type II collagen, T2CM, MMP-1/13-mediated degradation of type II collagen, C10C, cathepsin-K mediated degradation of type X collagen CRPM, C-reactive protein metabolite, PROM, MMP-1 and MMP-13-mediated degradation of prolargin, VICM, citrullinated and MMP-degraded vimentin, PRO-C2, pro-peptide of type II collagen, PRO-C3, pro-peptide of type III collagen, PRO-C6, type VI alpha-3 chain collagen.
